# Chromosome-Scale Genome Assembly and Characterization of Top-Quality Japanese Green Tea Cultivar ‘Seimei’

**DOI:** 10.1093/pcp/pcae060

**Published:** 2024-05-27

**Authors:** Yoshihiro Kawahara, Junichi Tanaka, Kazuhiro Takayama, Toshiyuki Wako, Akiko Ogino, Shuya Yamashita, Fumiya Taniguchi

**Affiliations:** Research Center for Advanced Analysis, NARO, Tsukuba, 305-8602 Japan; Institute of Crop Science, NARO, Tsukuba, 305-8518 Japan; Institute of Fruit Tree and Tea Science, NARO, Tsukuba, 305-8605 Japan; Institute of Crop Science, NARO, Tsukuba, 305-8518 Japan; Institute of Fruit Tree and Tea Science, NARO, Tsukuba, 305-8605 Japan; Institute of Fruit Tree and Tea Science, NARO, Tsukuba, 305-8605 Japan; Institute of Fruit Tree and Tea Science, NARO, Tsukuba, 305-8605 Japan

**Keywords:** Genome assembly, Genome diversity, Hi-C, Japanese green tea, PacBio HiFi, ‘Seimei’

## Abstract

Japanese green tea, an essential beverage in Japanese culture, is characterized by the initial steaming of freshly harvested leaves during production. This process efficiently inactivates endogenous enzymes such as polyphenol oxidases, resulting in the production of sencha, gyokuro and matcha that preserves the vibrant green color of young leaves. Although genome sequences of several tea cultivars and germplasms have been published, no reference genome sequences are available for Japanese green tea cultivars. Here, we constructed a reference genome sequence of the cultivar ‘Seimei’, which is used to produce high-quality Japanese green tea. Using the PacBio HiFi and Hi-C technologies for chromosome-scale genome assembly, we obtained 15 chromosome sequences with a total genome size of 3.1 Gb and an N50 of 214.9 Mb. By analyzing the genomic diversity of 23 Japanese tea cultivars and lines, including the leading green tea cultivars ‘Yabukita’ and ‘Saemidori’, it was revealed that several candidate genes could be related to the characteristics of Japanese green tea. The reference genome of ‘Seimei’ and information on genomic diversity of Japanese green tea cultivars should provide crucial information for effective breeding of such cultivars in the future.

## Introduction

Tea (*Camellia sinensis*) is one of the world’s most important beverage crops, along with coffee, and tea consumption is deeply ingrained in food cultures around the world. In recent years, with the growing worldwide appreciation of Japanese food, the interest in Japanese green tea, an indispensable beverage in Japanese cuisine, has been increasing. Tea has a relatively large genome among plants, 3 Gb, almost the same size as that of humans (Xia et al. 2020b). Black tea, green tea and semi-fermented teas such as oolong tea are all produced from the young shoots and leaves of *C. sinensis*. For each type of tea, suitable cultivars have been bred around the world. Japanese green tea includes sencha, gyokuro and matcha, which are characterized by bright green color; the color of young shoots and leaves is retained by efficiently deactivating enzymes such as polyphenol oxidases (PPOs) by first steaming the fresh leaves (Takeda 2007, Tanaka 2012, Qin et al. 2022). Japanese green tea is also used in traditional Japanese water-extracted and water-suspended powder beverages, as well as Western-style beverages such as matcha lattés. As a powdered food ingredient that confers bright green color, it is widely used in Japanese sweets, Western cookies and ice cream. Harvested young shoots and leaves of Japanese green tea cultivars tend to preserve brighter green color when processed into tea and tea water than those of the cultivars intended for different uses, even if they grow under the same conditions, and contain fewer catechins and more free amino acids (Ueno et al. 1979). This is probably the result of repeated phenotype-based selection during the breeding history of Japanese green tea.

‘Yabukita’ is a dominant green tea cultivar that has long been popular in Japan. The first turning point in the breeding of Japanese green tea cultivars was ‘Saemidori’, which was registered in 1991. This cultivar is highly regarded for its excellent color of processed tea leaves and infusion, strong umami taste and slight astringency, and is cultivated mainly in the southern part of Japan (Takeda 2006). The next major turning point was ‘Seimei’, registered in 2020, which further improved the quality of green tea, making it superior to ‘Saemidori’ (Yoshida et al. 2018). ‘Seimei’ produces high-quality Japanese green tea and has a bright green color of processed leaves and infusion, as well as a lower catechin content and a higher amino acid content than those of ‘Yabukita’ and ‘Saemidori’. These characteristics are likely the result of the accumulation of the alleles suitable for Japanese green tea during multiple generations of breeding and selection. Understanding which genomic regions and genes have been selected should provide important insights into further breeding of high-quality Japanese green tea cultivars. Elucidation of the mechanisms by which the combinations of alleles contribute to the above characteristics should further our understanding of how crops could be improved in response to human taste preferences.

In recent years, with the rapid progress of genome sequencing, genomic information has come to play an important role in plant breeding. Prediction of promising cross combinations is now possible on the basis of the results of quantitative trait locus (QTL) analysis, genome-wide association studies (GWAS), screening based on DNA markers and marker-assisted selection. In genome and variant analyses using high-throughput sequencing, a high-quality reference genome sequence is required to accurately detect differences in nucleotide sequences. To detect variants in a population with high accuracy, a reference genome sequence of a closely related cultivar or germplasm is necessary. Although several chromosome-level reference genome sequences of tea have been published (Wang et al. 2020, Xia et al. 2020a, Zhang et al. 2020a, 2020b, Lin et al. 2022, Chen et al. 2023), no such sequences are available for Japanese green tea cultivars.

Here, we have determined a chromosome-scale high-quality reference genome sequence of the Japanese green tea cultivar ‘Seimei’ (2*n* = 30) using the PacBio HiFi technology, which can produce high-quality long reads with average lengths >10 kb and accuracies greater than 99.5% (Hon et al. 2020). Whole-genome resequencing of ‘Seimei’ and other high-quality Japanese cultivars revealed several candidate genes and regions contributing to the characteristic traits. We also clarified the correspondence of the assembled chromosome sequences and the reference linkage map (Taniguchi et al. 2012b). We expect that the reference genome sequence and gene annotation of this representative of Japanese green tea cultivars will provide valuable information for future breeding of Japanese green tea cultivars.

## Results

### Genome assembly of ‘Seimei’

**Fig. 1:**
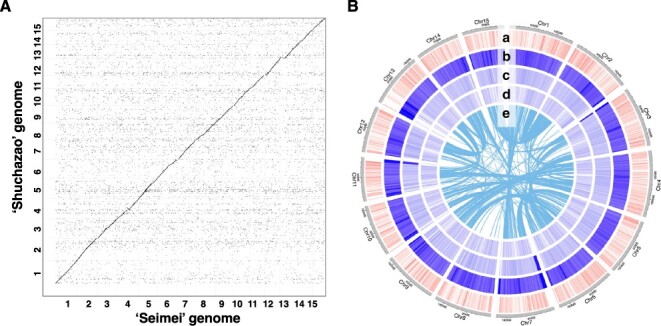
Structure and features of the ‘Seimei’ genome. (A) Dotplot between the ‘Seimei’ and ‘Shuchazao’ reference genomes. (B) A Circos diagram; tracks from outer to inner: a) gene density (frequency of genes per 1 Mb), b) repeat density (frequency of all types of repeats per 1 Mb), c) LTR/Gypsy density (frequency of LTR/Gypsy-type repeats per 1 Mb), d) LTR/Copia density (frequency of LTR/Copia-type repeats per 1 Mb). In the inner circle (e), colinear blocks between chromosomes are represented by connecting lines.

After filtering of the PacBio HiFi reads of ‘Seimei’ (**Supplementary Table S1**), approximately 123.5 Gb (41.2× coverage of the genome) of high-quality reads was assembled into a 3.2-Gb genome sequence consisting of 425 contigs. Using Hi-C data after misassembly correction, the contigs were assembled into a total of 413 scaffold sequences (3.2 Gb in total; N50 = 203.2 Mb). A dotplot between the 413 scaffolds and the reference genome of the Chinese tea cultivar ‘Shuchazao’ suggested that each chromosome was entirely covered by one or two large scaffold sequences (**Supplementary Fig. S1**). To obtain a chromosome-scale genome assembly, we used a reference-guided contig ordering and orienting tool to align 125 scaffold sequences and assemble them into 15 chromosome sequences (3.1 Gb in total; N50 = 214.9 Mb) (**Table 1**). After exclusion of sequences derived from chloroplast and mitochondrial genomes, 166 unanchored scaffolds remained (86.4 Mb in total; N50 = 16.5 Mb) (**Supplementary Table S2**). We named this genome sequence ‘Seimei v. 1.2’ and used it as the reference genome of ‘Seimei’. Although the genomic structure was generally consistent between ‘Seimei’ and ‘Shuchazao’, several small structural variations were found. Compared with the ‘Shuchazao’ genome, additional sequences were present at the ends of some chromosomes (e.g. Chrs. 7, 10, 11, 12 and 13) in the ‘Seimei’ genome, likely owing to a more precise assembly of the telomeric regions in our study (**Fig. 1A**). The complete Benchmarking Universal Single-Copy Orthologs (BUSCO) score of the ‘Seimei’ genome was 94.8%, of which 88.4% was single-copy BUSCOs. The genome assembly of ‘Seimei’ showed higher contiguity and BUSCO completeness than previously published tea genomes (**Table 1** and **Supplementary Table S3**; Zhang et al. 2020a, 2020b, Wang et al. 2020, Lin et al. 2022). The ‘Seimei’ genome had a long-terminal repeat (LTR) assembly index (LAI) of 14.2, which meets the criteria for a reference-quality assembly (10 ≤ LAI < 20) (Ou et al. 2018). It was also comparable to other published tea genomes assembled with long-read sequences (Wang et al. 2020, Xia et al. 2020a, Zhang et al. 2020a, Lin et al. 2022).

**Table 1:** Statistics of the ‘Seimei’ genome assembly in comparison with the published reference genomes

	Seimei	Shuchazao	Longjing 43
Reference	This study	Xia et al. 2020a	Wang et al. 2020
Assemble methods	PacBio HiFi, HiC	PacBio, HiC, Illumina	PacBio, HiC, 10X, BioNano
Genome size (Gb)	3.2	2.9	3.3
Size of 15 chromosomes (Gb)	3.1	2.6	2.3
Number of scaffolds	181	1,333	30,544
Scaffold N50 (Mb)	214.9	167.1	143.9
Non-ATGC bases (Mb)	0.2	0.6	0.7
Number of gaps	293	5,698	7,056
GC content (%)	38.8	38.3	38.7
LAI	14.2	15.5	12.1
BUSCO (Genome)			
Complete (Single, Dup.)	94.8% (88.4%, 6.4%)	92.0% (69.5%, 22.5%)	88.9% (82.0%, 6.9%)
Fragmented	2.0%	2.7%	3.5%
Missing	3.2%	5.3%	7.6%

To evaluate the accuracy of the assembly, we investigated the relationship between the ‘Seimei’ genome sequence and linkage maps using 297 simple sequence repeat (SSR) markers, including published (Taniguchi et al. 2012b) and new ones. Of these, 254 markers were uniquely mapped to the genome, and 243 (95.7%) markers were consistently assigned to the corresponding linkage group in the genetic map and chromosome in the assembly (**Supplementary Fig. S2 and Supplementary Table S4**). The collinearity of the genetic map and the ‘Seimei’ genome had a Pearson’s correlation coefficient (*r*) of 0.94, indicating the high contiguity and accuracy of the ‘Seimei’ genome assembly. No markers mapped to unanchored scaffolds, and none of these scaffolds could be incorporated into the chromosome sequences.

### Annotation of repetitive sequences and protein-coding genes in the ‘Seimei’ genome

Using a repeat library based on the ‘Seimei’ and two previously published tea genomes, we identified 79.4% of the ‘Seimei’ genome as repetitive sequences. This proportion was similar to those of published tea genomes (**Table 2** and **Supplementary Table S5**). Among the repeat sequences, 35% were derived from retroelements, the majority of which were LTR retrotransposons. The most common type of these was LTR/Gypsy (28.1%), followed by LTR/Copia (4.9%). These repeat sequences were distributed throughout the genome, with high proportions in the telomeric regions of some chromosomes (**Fig. 1B**). The proportion of repetitive sequences was lower in chromosome sequences (79.1%) than in scaffold sequences (89.8%) (**Supplementary Table S2**).

**Table 2:** Length of repetitive sequences in the ‘Seimei’ genome

	Seimei		Shuchazao		Longjing 43	
	Length (Mb)	%	Length (Mb)	%	Length (Mb)	%
Total repeats	2505.5	79.4	2247.4	76.7	2604.1	79.9
Retroelements	1106.8	35.1	1016.4	34.6	1199.5	36.8
SINEs	2.1	0.1	1.7	0.1	1.9	0.1
LINEs	39.9	1.3	40.5	1.4	40.5	1.2
LTR elements	1064.9	33.7	974.2	33.2	1157.1	35.5
Ty1/Copia	155.8	4.9	164.2	5.6	171.5	5.3
Gypsy/DIRS1	887.6	28.1	789.2	26.9	964.0	29.6
DNA transposons	57.9	1.8	59.9	2.0	60.7	1.9
Rolling circles	9.0	0.3	8.8	0.3	8.8	0.3
Unclassified	1310.4	41.5	1149.2	39.1	1320.5	40.5
Total interspersed repeats	2475.0	78.4	2225.5	75.7	2580.8	79.2
Small RNA	10.1	0.3	1.5	0.1	1.9	0.1
Satellites	12.1	0.4	12.3	0.4	13.3	0.4
Simple repeats	0.3	0.0	0.3	0.0	0.3	0.0

To predict gene models, we performed strand-specific RNA sequencing (RNA-Seq) and PacBio isoform sequencing (IsoSeq) using young shoots, mature leaves and roots (**Supplementary Table S1**). By combining gene models constructed by mapping of IsoSeq reads and *ab initio* gene prediction with RNA-Seq data, we obtained 55,235 protein-coding loci corresponding to 91,390 transcripts (**Table 3**). The BUSCO completeness of the ‘Seimei’ gene models was much higher (97.5%) than that of the gene models in previously published tea genomes [e.g. 89.7% for ‘Shuchazao’ and 84.2% for ‘Longjing 43 (LJ43)’; **Table 3** and **Supplementary Table S3**]. To functionally annotate the gene models, we estimated gene abundance using RNA-Seq data, homology searches against the databases of known proteins (SwissProt, TrEMBL and TPIA), a functional domain search against InterPro and assignment of gene ontology (GO) and Kyoto Encyclopedia of Genes and Genomes (KEGG) orthology (KO) terms. Overall, 26,935 genes (48.8%) were expressed [transcripts per million (TPM) > 1] in at least one of the three tissues (**Supplementary Fig. S3**). Of those, 11,645 genes (43.2%) were expressed in all three tissues. Of the 18,388 genes expressed in young shoots, 1,532 genes (5.7%) were young-shoot specific. The products of 52,366 genes (94.8%) had homologs in the UniProt database when the entire database was searched, and the products of 51,324 genes (92.9%) had homologs when the search was limited to *Camellia* proteins (**Supplementary Table S6**). The products of 31,142 genes (56.4%) had homologs in the SwissProt curated protein database (**Supplementary Table S6**). The products of 38,955 genes had at least one InterPro functional domain; InterProScan assigned GO terms to 27,970 genes (50.6%), and BlastKOALA assigned KO terms to 12,577 genes (22.8%).

**Table 3:** Gene annotation of tea cultivars

	Seimei (v. 1.2, all transcripts)	Seimei (v. 1.2, only primary transcripts)	Shuchazao	Longjing 43
Number of loci	55,235	55,235	50,524	33,556
Number of transcripts	91,390	55,235	50,524	33,556
BUSCO (protein)				
Complete (Single, Dups.)	97.5% (52.3%, 45.2%)	97.4% (88.5%, 8.9%)	89.7% (55.1%, 34.6%)	84.2% (77.1%, 7.1%)
Fragmented	1.5%	1.5%	4.5%	7.3%
Missing	1.0%	1.1%	5.8%	8.5%

To facilitate further gene-based analyses, a representative ‘primary transcript’ was selected for each locus. When only the primary transcripts were used, the BUSCO duplication rate was significantly lower, but there was little effect on the completeness of the annotation (**Table 3**).

### Genome diversity in ‘Seimei’ and other tea varieties

To infer the phylogenetic relationships of Japanese green tea, including ‘Seimei’ and other cultivars, we analyzed whole-genome resequencing data and obtained genome-wide variation data for 26 tea varieties (**Supplementary Table S7**). In addition to the genome assembly and gene annotation of ‘Seimei’, information on single nucleotide polymorphisms (SNPs) and indels between the genomes of ‘Seimei’ and each variety could be obtained through the multiple-genome browser TASUKE+ (Kumagai et al. 2019), which displays the distribution of the variants in any genomic region together with functional gene annotation.

**Fig. 2:**
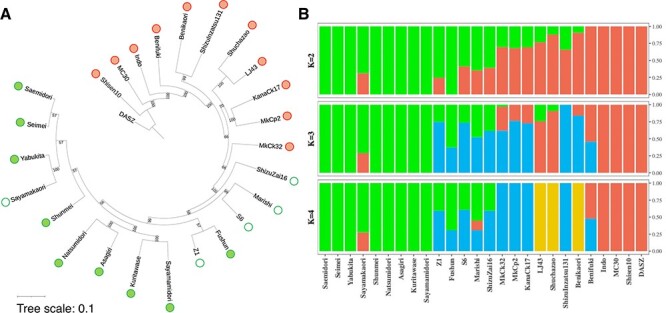
Phylogenetic and population genetic analyses of 26 tea varieties. (A) A maximum-likelihood phylogenetic tree. Green circles, Japanese green tea cultivars; green open circles, admixture of Japanese green tea cultivars and other varieties; red circles, other tea varieties. (B) Population structure inferred by ADMIXTURE with 22,510,195 pruned variant sites; *K *= 2 was the best inferred *K* value (i.e. lowest cross-validation errors).

The inferred maximum likelihood phylogenetic tree indicated that 14 cultivars and germplasms were closely related and formed a single clade with high bootstrap support (**Fig. 2A**). To investigate the genetic relationship among the 26 varieties, we used principal component analysis (PCA) with 33,616,569 bi-allelic and unlinked SNPs (**Supplementary Fig. S4**). In the PCA, germplasms and cultivars of Japanese green tea formed a cluster, and the Chinese cultivars (Shuchazao and LJ43) and wild tea (DASZ) were distantly related to the Japanese varieties. Analysis of the population structure of the 26 tea genomes with the optimal *K* of 2 suggested the existence of two groups: Japanese green tea and other varieties, the latter consisting of foreign varieties, their hybrids with Japanese varieties and a wild accession (**Fig. 2B**). Since the genomes of five Japanese green tea varieties (‘Sayamakaori’, ‘Z1’,‘S6’, ‘Marishi’ and ‘ShizuZai16’) contained various proportions of non-Japanese green tea-type genomic components, we defined them as an admixture of Japanese green tea and other varieties (**Supplementary Table S7**). In the PCA, these five varieties were located a short distance away from other Japanese green tea cultivars (**Supplementary Fig. S4**). Increasing *K* to 3 or 4 divided the remaining 12 other varieties (‘MakCk32’, ‘MakCp2’, ‘KanaCk17’, ‘LJ43’, ‘Shuchazao’, ‘ShizuInzatsu131’, ‘Benikaori’, ‘Benifuki’, ‘Indo’, ‘MC30’, ‘Shisen10’ and DASZ) into multiple groups, including Chinese tea (‘LJ43’ and ‘Shuchazao’) and hybrids of var. *sinensis* and var. *assamica* (‘Benikaori’, ‘Benifuki’ and ‘Indo’ are suitable for black tea).

**Fig. 3:**
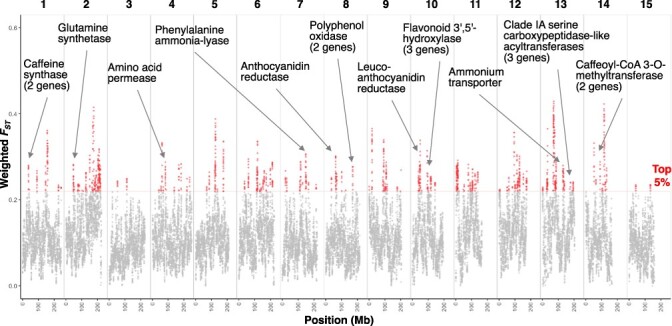
Population differentiation between Japanese green tea cultivars and other tea varieties. Weighted *F*_ST_ values were calculated and plotted for each 1-Mb window along chromosomes. Windows with high *F*_ST_ values (top 5%) are shown in red. Several agronomically important genes in the high-*F*_ST_ regions are indicated by arrows.

To explore the genomic regions and genes subject to selection pressure in the Japanese green tea cultivars, we defined a group of nine Japanese cultivars (‘Seimei’, ‘Saemidori’, ‘Yabukita’, ‘Shunmei’, ‘Natsumidori’, ‘Asagiri’, ‘Kuritawase’, ‘Sayamamidori’ and ‘Fushun’) and a group of 11 other varieties (‘MakCk32’, ‘MakCp2’, ‘KanaCk17’, ‘LJ43’, ‘Shuchazao’, ‘ShizuInzatsu131’, ‘Benikaori’, ‘Benifuki’, ‘Indo’, ‘MC30’ and ‘Shisen10’) and performed population genetic analyses. We estimated nucleotide diversity and *F*_ST_ values between the two groups in each 1-Mb window. The overall nucleotide diversity was significantly lower for the Japanese cultivars than for the other varieties (**Supplementary Fig. S5**). Over the entire genome, regions totaling approximately 0.16 Gb (4.9%) and containing 5,003 genes had higher *F*_ST_ values (top 5%) and lower nucleotide diversity in the Japanese green tea cultivars than in the other varieties (**Fig. 3**, **Supplementary Fig. S6 and Supplementary Table S8**), suggesting that these regions and genes were subjected to selection pressure during the breeding of Japanese green tea cultivars. From these 5,003 genes, we picked up 40 genes related to the quality of Japanese green tea by keyword search of annotation information, such as genes encoding PPOs and enzymes involved in catechin and caffeine synthesis (**Table 4**).

**Table 4:** Characteristics of the genes of Japanese (JP) green tea cultivars subject to artificial selection

Gene ID	Location	Description	TPM (Leaf)	TPM (Bud)	TPM (Root)	*F*_ST_	Pi (JP)	Pi (Others)	Reference
PPOs									
CsSME08G250700	Chr8:170,186,686–170,189,610	PPO; *CsPPO3* (MK977644)	0.00	0.47	11.84	0.233	0.006	0.010	Yu et al. 2020
CsSME08G251100	Chr8:170,467,597–170,471,672	PPO; *CsPPO1* (MK977642)	1.70	3.26	8.03	0.278	0.005	0.010	Yu et al. 2020
Catechin biosynthesis									
CsSME10G160100	Chr10:115,924,470–115,926,391	Flavonoid 3ʹ,5ʹ-hydroxylase	0.00	0.00	1.17	0.254	0.004	0.012	
CsSME10G160200	Chr10:115,929,555–115,930,082	Flavonoid 3ʹ,5ʹ-hydroxylase	5.04	0.00	0.00	0.254	0.004	0.012	
CsSME10G160400	Chr10:116,137,586–116,142,948	Flavonoid 3ʹ,5ʹ-hydroxylase	0.00	0.00	33.78	0.254	0.004	0.012	
CsSME07G265100	Chr7:149,081,465–149,088,164	Phenylalanine ammonia-lyase; *CsPALe* (KY615672)	2.62	0.95	51.58	0.255	0.006	0.010	Wu et al. 2017
CsSME02G128100	Chr2:46,683,327–46,683,545	4-Coumarate coenzyme A ligase	0.00	0.00	0.53	0.238	0.008	0.012	
CsSME02G133700	Chr2:48,992,210–48,994,417	4-Coumarate coenzyme A ligase	0.19	4.08	0.10	0.240	0.007	0.013	
CsSME02G229500	Chr2:86,105,384–86,124,120	4-Coumarate coenzyme A ligase	3.40	2.93	2.08	0.234	0.006	0.012	
CsSME02G379100	Chr2:203,220,516–203,229,556	4-Coumarate coenzyme A ligase; *Cs4CL1* (KY615680)	0.25	3.71	188.84	0.221	0.007	0.011	Li et al. 2022
CsSME09G003000	Chr9:1,654,209–1,654,601	4-Coumarate coenzyme A ligase	0.00	0.00	0.13	0.223	0.004	0.008	
CsSME14G226600	Chr14:91,541,015–91,544,640	Caffeoyl-CoA 3-O-methyltransferase; *CsCCoAOMT4*	18.03	24.25	17.65	0.255	0.007	0.010	Lin et al. 2021
CsSME14G226700	Chr14:91,609,944–91,615,496	Caffeoyl-CoA 3-O-methyltransferase	5.32	46.90	72.47	0.238	0.008	0.010	
CsSME08G113800	Chr8:61,612,366–61,617,252	Anthocyanidin reductase; *CsANR1* (GU992402)	6.44	35.71	20.15	0.290	0.004	0.009	Pang et al. 2013
CsSME13G256900	Chr13:173,365,813–173,385,970	Clade IA SCPL-IA; *CsSCPL2*	0.00	0.00	15.82	0.225	0.004	0.007	Zhao et al. 2023
CsSME13G259400	Chr13:173,870,095–173,876,770	Clade IA SCPL-IAs; *CsSCPL1*	0.00	1.79	0.10	0.225	0.003	0.006	Zhao et al. 2023
CsSME13G259500	Chr13:173,888,801–173,911,008	Clade IA SCPL-IAs; *CsSCPL2*	1.97	1.00	11.97	0.225	0.003	0.006	Zhao et al. 2023
CsSME08G063100	Chr8:35,704,661–35,713,652	Flavonol synthase	1.31	0.00	52.23	0.251	0.003	0.008	
CsSME10G066300	Chr10:41,313,170–41,328,004	Leucoanthocyanidin reductase; *CsLARb* (KY615700)	0.00	6.31	0.00	0.240	0.006	0.010	Wang et al. 2018
CsSME01G418400	Chr1:226,308,260–226,314,513	Chorismate mutase	2.39	0.74	8.83	0.222	0.004	0.007	
CsSME10G163100	Chr10:117,323,412–117,335,741	Tryptophan synthase	0.03	0.00	6.22	0.245	0.004	0.010	
CsSME06G108300	Chr6:36,333,905–36,342,687	Tyrosine aminotransferase	0.77	0.22	1.38	0.243	0.005	0.009	
Nitrogen assimilation/amino acid metabolism									
CsSME02G381700	Chr2:204,118,537–204,139,839	Aspartate kinase	0.00	0.00	7.27	0.304	0.006	0.011	
CsSME01G420100	Chr1:226,563,952–226,569,518	Cystathionine gamma synthase	16.22	27.15	7.57	0.229	0.004	0.007	
CsSME10G160800	Chr10:116,251,734–116,267,048	Acetylglutamate kinase	1.78	0.99	6.94	0.254	0.004	0.012	
CsSME02G140600	Chr2:51,865,929–51,871,693	Glutamine synthetase; *CsGS1.2* (AB115184.1, KY649470)	16.01	33.94	25.08	0.230	0.006	0.011	
CsSME07G034100	Chr7:28,647,464–28,650,405	Ornithine carbamoyltransferase	4.14	3.72	4.16	0.240	0.005	0.008	
CsSME13G293000	Chr13:187,012,588–187,014,466	Polyamine oxidase	14.90	1.59	2.65	0.221	0.003	0.005	
CsSME04G171000	Chr4:82,564,623–82,567,827	Amino acid permease; *CsAAP3*	0.00	7.44	56.76	0.288	0.009	0.015	Dong et al. 2020
CsSME04G378300	Chr4:219,593,400–219,598,214	Amino acid permease; *CsAAP8*	0.00	0.00	3.49	0.249	0.006	0.010	Dong et al. 2020
CsSME04G378700	Chr4:219,686,626–219,692,239	Amino acid permease; *CsAAP9*	0.80	0.58	1.92	0.230	0.006	0.010	Dong et al. 2020
CsSME06G419400	Chr6:209,783,235–209,784,663	Amino acid permease	0.00	1.01	0.73	0.287	0.004	0.007	
CsSME10G085300	Chr10:51,148,886–51,155,413	Amino acid permease	5.17	1.39	10.61	0.250	0.005	0.008	
CsSME13G148200	Chr13:133,382,578–133,385,150	Ammonium transporter; *AMT1.4* (MK905205)	0.00	0.98	0.00	0.228	0.006	0.010	
CsSME10G060000	Chr10:38,681,456–38,684,775	Arginine decarboxylase	4.52	2.49	29.15	0.251	0.005	0.010	
Caffeine biosynthesis									
CsSME01G095600	Chr1:36,330,084–36,331,406	Caffeine synthase	0.00	0.11	0.00	0.224	0.006	0.009	
CsSME01G095700	Chr1:36,474,905–36,481,624	Caffeine synthase; *TCS1* (AB031280)	48.57	259.62	190.83	0.245	0.006	0.009	Yoneyama et al. 2006
CsSME01G095900	Chr1:36,706,249–36,715,758	Caffeine synthase; *TCS2* (AB031281)	0.06	17.73	3.06	0.280	0.005	0.009	Yoneyama et al. 2006
CsSME01G096100	Chr1:36,939,299–36,940,534	Caffeine synthase	0.00	0.09	0.00	0.280	0.005	0.010	
CsSME01G096500	Chr1:37,672,128–37,676,916	Caffeine synthase	0.00	0.00	2.56	0.280	0.005	0.010	

### Genes characteristic for Japanese green tea cultivars

**Fig. 4:**
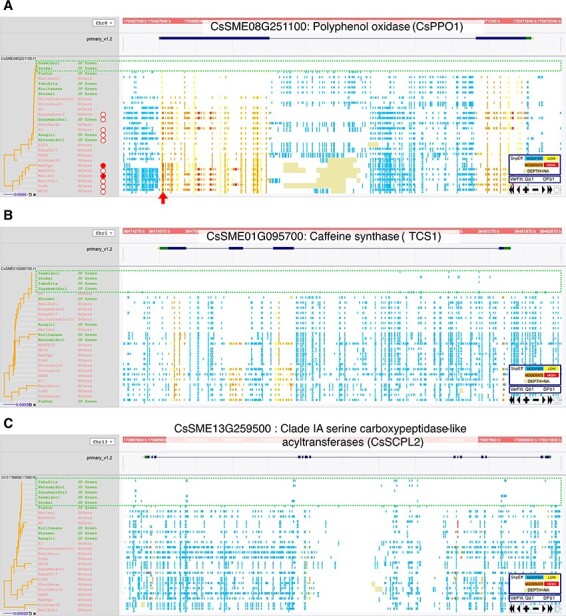
Nucleotide variations among 26 tea varieties in the genic regions related to the characteristic traits of Japanese green tea cultivars. (A) PPO (*CsPPO1*), (B) caffeine synthase (*CsTCS1*) and (C) Clade IA SCPL-IA (*CsSCPL2*). SNPs and indels vs. the ‘Seimei’ genome were visualized in TASUKE + . Each variant is colored according to its effect on the protein sequence (from low to high). Green dotted rectangles indicate genomic regions fixed in homozygous ‘Seimei’-type genotypes. A red arrow indicates a candidate functional mutation. Open and filled red circles indicate varieties heterozygous and homozygous for the mutation, respectively.

In the first step of the green tea production process, harvested fresh leaves are roasted or steamed to deactivate enzymes in order to preserve the green color. The main target enzymes are PPOs, which oxidize catechins and chlorophylls (Tang et al. 2023). Oxidation of chlorophylls in tea leaves leads to their degradation and to the loss of the bright green color of the leaves. Two PPO genes were found among the 40 genes that were subjected to selection pressure and are expressed in young shoots, mature leaves and roots. Nine varieties had heterozygous and two varieties had homozygous alleles of *CsPPO1* (CsSME08G251100) with a premature stop codon immediately after the translation start site (**Fig. 4A**). In comparison with the ‘Seimei’ sequence, 20 of the 26 varieties had heterozygous alleles of *CsPPO3* (CsSME08G250700) with a three-amino acid deletion that overlapped with a di-copper center-containing domain encoded by the first exon, but no variant affecting the amino acid sequence was observed in ‘Saemidori’, the pollen parent of ‘Seimei’ with bright green leaves (**Supplementary Fig. S7A**).

**Fig. 5:**
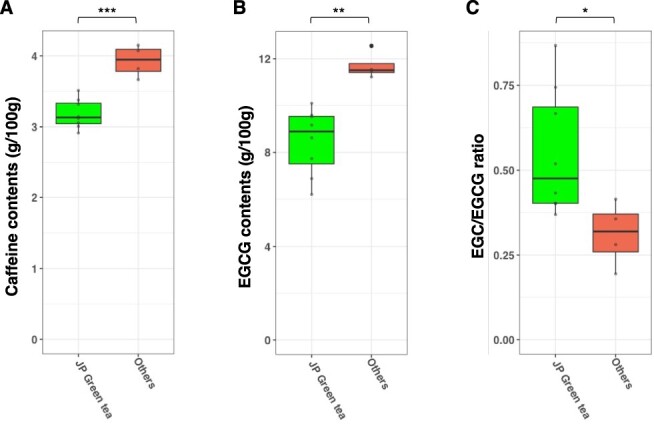
Comparisons of (A) caffeine contents, (B) EGC and EGCG contents and (C) the EGC/EGCG ratio between Japanese (JP) green tea cultivars and other tea varieties. JP green tea: Seimei, Saemidori, Fushun, Shunmei, Yabukita, Sayamamidori, Natsumidori and Asagiri. Others: Benifuki, Benikaori, ShizuInzatsu131 and Indo. Significant differences: ***P < 0.001, **P < 0.01, *P < 0.05 (Student’s *t*-test using one-tailed distributions).

Genetic diversity of the caffeine synthase gene *CsTCS1* (CsSME01G095700) was extremely low in the Japanese cultivars ‘Seimei’, ‘Saemidori’, ‘Sayamamidori’ and ‘Yabukita’ (**Fig. 4B**). Japanese cultivars had significantly lower caffeine content than the other varieties (**Fig. 5A**).

A total of 25 Clade IA serine carboxypeptidase-like acyltransferase (SCPL-IA) genes were found in the ‘Seimei’ genome. SCPL-IAs are involved in the synthesis of catechin gallates (Yao et al. 2022, Zhao et al. 2023). Biases in genetic diversity were also observed in three *SCPL-IA* genes (**Fig. 4C**, **Supplementary Fig. S7B and C**). ‘Seimei’ and several other Japanese green tea cultivars had particularly few mutations and were homozygous for the same alleles. Epigallocatechin gallate (EGCG) is the major astringent component of tea. Epigallocatechin (EGC) is less astringent than EGCG (Ye et al. 2022), and the ratio of EGC to EGCG is a good indicator of tea astringency. The content of EGCG was significantly lower, and the EGC/EGCG ratio was significantly higher in Japanese green tea cultivars than in the other varieties (**Fig. 5B and C**).

It is noteworthy that almost all the nucleotide sites in these candidate genes had the same genotype and were fixed to a homozygous in both ‘Seimei’ and its pollen parent ‘Saemidori’. This might suggest that these candidate genes have been subjected to artificial selection during the breeding process of these superior cultivars.

## Discussion

### High-quality reference genome and gene annotation of Japanese green tea cultivars

High-quality reference genome sequences can be valuable for efficient molecular marker development and understanding of the molecular basis of characteristic traits. The available reference genome sequences of Chinese tea cultivars (Wang et al. 2020, Xia et al. 2020a) are not reliable as a reference for marker development or molecular biological studies of Japanese green tea cultivars. Here, we constructed a highly accurate chromosome-scale reference genome and gene annotation of the top-quality Japanese green tea cultivar ‘Seimei’. Its sequence quality was equal to or better than that of previously determined genome sequences of Chinese tea cultivars and wild tea (Wang et al. 2020, Zhang et al. 2020a, 2020b, Lin et al. 2022, Chen et al. 2023). The gene models of ‘Seimei’ were determined by the state-of-the-art long- and short-read transcriptome sequencing technologies and were more complete than those in the previously published tea genomes. The high-quality complete reference genome sequence and gene annotation will provide crucial information for future molecular breeding of Japanese green tea cultivars.

### Toward accelerated breeding of Japanese green tea based on genomic information

We clarified the correspondence between the previously constructed linkage map (Taniguchi et al. 2012b) and genetic markers in the genome sequence. This relationship paves the way for gene identification from the already known QTLs. The identified genes and information on their polymorphism will be useful for the development of selection markers to be used in molecular breeding programs (Wang et al. 2023).

Although high quality of green tea is the most important breeding goal, there are many other desirable breeding traits of tea cultivars, such as pest resistance, high yield and early and late harvest. In the last two decades, Japanese green tea breeding has been directed toward methods that allow efficient accumulation of useful alleles from a wide range of breeding materials by combining phenotype-based selection with technologies based on early selection with molecular markers to shorten the time required for one generation (Tanaka 2006a, 2012). As a basis for such methods, molecular markers and high-density linkage maps have been developed to estimate genotypes at specific locations in the tea genome (Taniguchi et al. 2012b). Molecular markers have been used to select for resistance to mulberry scale (*Pseudaulacaspis pentagona*) (Tanaka 2006b). We are developing molecular markers for other traits, but designing markers at precise locations in the genome is still a challenging task without accurate information on variants among breeding materials.

With the availability of whole-genome resequencing data, it has recently become easier to obtain genome-wide diversity information for diverse varieties. To comprehensively characterize features common to the genomes of ‘Seimei’ and other Japanese cultivars, we sequenced 23 tea genomes, including those of superior Japanese green tea cultivars, breeding lines and germplasms useful for Japanese tea breeding. Whole-genome variant detection was performed after adding available genome sequence data of three varieties (‘Shuchazao’, ‘LJ43’ and ‘DASZ’). The resulting information on variants is publicly available through the multiple genome browser TASUKE+, which allows users to explore SNPs and indels around any gene or genomic region in the 26 varieties. This information can be used for molecular breeding and to find mutations that might affect gene function, classify the haplotypes of genes in each variety and efficiently design DNA markers to detect these mutations and to determine haplotypes.

‘Seimei’ has been actively used as an excellent breeding parent for the development of modern Japanese green tea cultivars, and many of its progeny populations have been deployed in the breeding programs in Japan. We expect the genetic markers that can be used to trace the inheritance of genome fragments of ‘Seimei’ to play an important role in selection breeding and genetic analysis of hybrid populations derived from crosses between ‘Seimei’ and other cultivars. The ‘Seimei’ genome and gene information will contribute to the further development of new cultivars of Japanese green tea with superior quality and better agronomic traits.

### Candidate genes involved in the establishment of green tea cultivars

Japanese green tea cultivars are known for their high tea-making quality, but the genetic factors that determine this quality are not well understood. Our population genetic analyses revealed several candidate genes that might have contributed to the development of the cultivars. The green color of leaves and infusions are very important for Japanese green teas. While high fermentability is a requirement for black tea cultivars, low fermentability is desirable for green tea cultivars. The biases in genetic diversity observed in PPO genes suggest that these genes (such as CsSME08G250700 and CsSME08G251100) were subjected to human selection pressure. Caffeine contents are lower in Japanese landraces than in foreign germplasms of *C. sinensis* var. *sinensis* and *C. sinensis* var. *assamica* (Takeda 2002, Yamashita et al. 2021). The low diversity of the caffeine synthase gene *TCS1* (CsSME01G095700) may be due to the selection of genotypes suitable for Japanese green tea. Among *SCPL-IA* genes, *SCPL2, 4* and *5* are involved in catechin galloylation, as suggested by gene expression and biochemical analyses, but the function of *SCPL1* remains unknown (Zhao et al. 2023). *SCPL-IA* genes other than those analyzed in this study are present in the ‘Seimei’ genome. The wide variation in catechin profiles among cultivars might be caused by differences in the functions and activities of *SCPL-IA* genes (**Fig. 5**). Further research, including biochemical analysis of CsSCPL1 and 2, is needed to provide new insights into the mechanisms of diversification of catechin profiles.

Here, we identified several candidate genes related to the quality of Japanese tea. To confirm these findings, it is necessary to identify the links between genotypes and phenotypes by population genetic analyses, such as GWAS, with a population consisting of diverse Japanese cultivars and germplasms, which would provide more phenotypic information. Furthermore, GWAS would make it possible to identify novel genes related to important traits and to develop new breeding and selection techniques by genomic prediction. ‘Saemidori’ and the further improved ‘Seimei’, which were the turning points in the breeding of Japanese green tea, particularly in terms of their superior green tea quality, have many homozygous regions in these candidate genes. This likely reflects the history of artificial selection in the breeding process. We expect that the identification of these candidate genes will provide important clues for the elucidation of the molecular mechanisms related to the quality of Japanese green tea.

## Materials and Methods

### Genome sequencing

New leaves of ‘Seimei’ were sampled at the Makurazaki Tea Research Station, Institute of Fruit and Tea Science, National Agriculture and Food Research Organization, Makurazaki, Kagoshima, Japan, when the new shoots of the first flush had stopped growing. High-molecular weight DNA from fresh leaves was extracted with the cetyltrimethylammonium bromide method (Doyle and Doyle 1987). A library was prepared using the SMRT-bell Express Template Kit 2.0 (Pacific Bioscience, Menlo Park, CA, USA). DNA concentration in the library was measured with a Qubit spectrophotometer, and size distribution was detected with a bioanalyzer. The Quantified library was sequenced on a PacBio Sequel II system (Pacific Bioscience). A Hi-C library was constructed with the Proximo Hi-C (Plant) Kit (Phase Genomics, Seattle, WA, USA) and sequenced on a NovaSeq 6000 system (Illumina Inc., San Diego, CA, USA).

### De novo genome assembly

Short and low-quality PacBio HiFi reads were discarded in Filtlong v. 0.2.0 software (https://github.com/rrwick/Filtlong) with the parameters—min_length 5000 and—min_mean_q 20. Filtered HiFi reads were assembled in hifiasm v. 0.16.1 with Hi-C data and the −z20 option (Cheng et al. 2021). To select high-quality Hi-C read pairs, which are informative for scaffolding, the ArimaGenomics mapping pipeline was used (https://github.com/ArimaGenomics/mapping_pipeline). Scaffolding of the assembled primary contigs was performed in SALSA v. 2.3 with the option ‘-m yes’ for assembly error correction (Ghurye et al. 2019). The genome assembly of *C. sinensis* var. *sinensis* cv. Shuchazao was downloaded from the TPIA database (http://tpia.teaplants.cn/) (Xia et al. 2019) and used as the reference genome in the pseudomolecule construction. Chromosome-scale sequences were constructed by anchoring the scaffolds on the 15 reference chromosomes in RagTag v. 2.1.0 (Alonge et al. 2022). To remove unanchored contigs, which were derived from organelle genomes, we used complete chloroplast (NC_061691.1) and mitochondrial (NC_043914.1) genome sequences in the RefSeq database as references. Unanchored contigs were aligned to the organelle genomes in minimap2 v. 2.24 (Li and Birol 2018). We removed the contigs meeting the criteria of mapping quality ≥20 and ≥90% of the contig aligned with <0.001 divergence. The assignment of chromosome numbers was based on the alignment with the ‘Shuchazao’ genome.

### Comparison with other reference genomes and genetic map

Previously reported tea reference genomes were obtained from the TPIA database. BUSCO v. 5.3.0 and the embryophyta_odb10 lineage dataset were used to assess the completeness of each genome assembly (Manni et al. 2021). Dotplot comparisons between the genome of ‘Seimei’ and other genomes were performed in minimap2 with the −x asm5 option, and the plots were drawn in dotPlotly (https://github.com/tpoorten/dotPlotly) with the −m 5000 and −q 10,000 options.

To assess the accuracy of the ‘Seimei’ genome assembly, we used an SSR-based linkage map, in which new SSR markers were added to the published genetic map (Taniguchi et al. 2012b) for collinearity analysis. The new SSR markers were developed by searching for SSR motifs with the microsatellite identification tool program (Thiel et al. 2003) among the expressed sequence tags of tea (Taniguchi et al. 2012a), and primers were designed using Primer3 (Untergasser et al. 2012). A total of 297 markers available on the genetic map were mapped to the genome assembly in BLASTN v. 2.2.31+ (Camacho et al. 2009) with default parameters. Markers that mapped to ≥2 positions were removed.

### Sequencing of ‘Seimei’ transcriptome

Total RNA was extracted from young shoots, mature leaves and roots for SMART-seq and from roots for IsoSeq with a NucleoSpin RNA Plant and Fungi Kit (Takara, Shiga, Japan). Total RNA was extracted from buds and leaves for IsoSeq with RNeasy Plant Mini kits (Qiagen, Hilden, Germany). Strand-specific SMART-Seq libraries were constructed with a SMART-Seq v. 4 Ultra Low Input RNA Kit for Sequencing (Takara) and sequenced on a NovaSeq 6000 system. For IsoSeq analysis, the NEBNext Single Cell/Low Input cDNA Synthesis & Amplification Module (New England Biolabs, Ipswich, MA, USA) and IsoSeq Express Oligo Kit (Pacific Biosciences) were used for reverse transcription and adapter ligation to the first-strand cDNA. Double-strand cDNA was synthesized by polymerase chain reaction (PCR) amplification, and libraries were prepared with a SMARTbell Express Template Prep Kit 2.0 (Pacific Biosciences). The libraries were sequenced on a Sequel II system (Pacific Biosciences).

### Transcriptome sequence analysis

IsoSeq reads were filtered and clustered in IsoSeq3 v. 3.8.0 (https://github.com/PacificBiosciences/IsoSeq). Full-length IsoSeq reads were aligned to the genome sequence in minimap2 v. 2.24. To obtain non-redundant isoforms, the cDNA_Cupcake v. 29.0.0 pipeline (https://github.com/Magdoll/cDNA_Cupcake) was used. Protein-coding regions were predicted for each transcript in TransDecoder v. 5.7.0 (https://github.com/TransDecoder/TransDecoder) with protein sequences in the plant division of SwissProt Release 2022_05 (https://www.uniprot.org/) and the Pfam database v. 35.0 (https://www.ebi.ac.uk/interpro/) as references. IsoSeq transcripts with complete open reading frames were used for gene annotation.

To remove adapters and low-quality sequences, the SMART-Seq reads were preprocessed in Trimmomatic v. 0.39 with the following parameters: adapters.fa:2:30:10 LEADING:15 TRAILING:15 SLIDINGWINDOW:10:15 MINLEN:100 (Bolger et al. 2014). The preprocessed reads were mapped to the ‘Seimei’ genome in HISAT2 v. 2.2.1 (Kim et al. 2019). Gene abundance was estimated in StringTie v. 2.2.1 (Pertea et al. 2015).

### Genome and gene annotation

In a singularity environment, Dfam TE Tools Container v. 1.6 was used for repeat masking in the genomes (https://github.com/Dfam-consortium/TETools). To construct a repeat library, RepeatModeler v. 2.0.3 (http://www.repeatmasker.org/ RepeatModeler) was used with three genome sequences of ‘Seimei’, ‘Shuchazao’ and ‘Longjing 43’. RepeatMasker v. 4.1.3 (http://www.repeatmasker.org/) was used to detect and mask repetitive regions in the reference genomes.

Protein-coding genes in the repeat-masked ‘Seimei’ genome were predicted by using the BRAKER3 pipeline v. 3.0.3 (Brůna et al. 2021). The SMART-Seq alignment and known *Camellia* protein sequences obtained from the UniProt database (Release 2022_05; UniProt Consortium, Bateman et al. 2023) were used as hints. Reference gene annotations of ‘Shuchazao’ (CSS_ChrLev_20200506) were downloaded from TPIA, mapped and transferred to the ‘Seimei’ genome in Liftoff v. 1.6.3 (Shumate et al. 2021). Gene models constructed by IsoSeq analysis, BRAKER3 and Liftoff were merged and redundant gene models were removed in gffread v. 0.12.7 (Pertea and Pertea 2020) and in-house scripts.

Functional domains and GO terms for each protein sequence were assigned with InterProScan v. 5.61–93.0 (Blum et al. 2021). Homology searches against protein databases (SwissProt, TrEMBL and the TPIA gene annotation) were performed with Diamond v. 2.1.6 (Buchfink et al. 2021). KEGG orthology terms were assigned to each protein sequence in BlastKOALA (Kanehisa et al. 2016).

### Whole-genome resequencing using short reads

DNA was extracted from leaves of 23 varieties with the DNeasy Plant Mini Kit (Qiagen) (**Supplementary Table S1**). Libraries were prepared with PCR-Free DNA Library Prep Kits according to the manufacturer’s protocol. Paired-end sequencing was performed with read lengths of 150 bp on NovaSeq 6000 (Illumina) and MGI DNBSEQ-G400 (MGI Tech Co., Ltd., Shenzhen, China) systems.

### Genome diversity analysis

Low-quality and adapter sequences were trimmed in Trimmomatic as in the transcriptome sequence analysis. Filtered paired-end reads were aligned to the ‘Seimei’ reference genome in bwa-mem2 v. 2.2.1 (Vasimuddin et al. 2019). PCR duplicates were removed in Picard v. 2.25.5 (https://broadinstitute.github.io/picard/), and variant calling was performed by HaplotypeCaller in GATK v. 4.2.6.1 (Van der Auwera and O’Connor 2020). Joint genotyping was performed with variant call data for 26 varieties in GenotypeGVCFs. Variants were filtered with VariantFiltration, and reliable variants were selected with SelectVariants in GATK with parameters QD < 2.0, MQ < 40.0, FS < 60.0, SOR > 3.0, MQRunkSum <−12.5, ReadPosRankSum <−8.0 for SNPs, and QD < 2.0, FS > 200.0, SOR > 10.0, ReadPosRunkSum <−20.0 for indels. All SNPs and indels among the 26 varieties were annotated in SnpEff v. 5.1 (Cingolani et al. 2012) with the gene annotations of ‘Seimei’ primary transcripts, and were published from TASUKE+ (v. 20,231,214; https://agrigenome.dna.affrc.go.jp/tasuke/Tea_Seimei/) (Kumagai et al. 2019).

Bi-allelic SNPs with MAF > 0.05 were extracted in vcftools v. 0.1.16 (Danecek et al. 2011). Phylogenetic relationships among the 26 varieties were inferred on the basis of 54,519,890 variant sites with the maximum-likelihood method in RAxML 8 with the model ‘GTR+ASC_LEWIS’ and 100× bootstrap tests (Stamatakis 2014). Nucleotide diversity and *F*_ST_ statistics were estimated with vcftools; nucleotide diversity was calculated for each 1-Mb window with a 100-kb overlap. Linked SNPs were pruned using PLINK (v. 1.9) with the parameters—indep-pairwise 100 5 0.8—and the population structure of the genomes was estimated in ADMIXTURE v. 1.3.0 (Alexander et al. 2009, Chang et al. 2015). Based on the same pruned SNP data, PCA was performed using PLINK (v. 1.9).

### Measurement of caffeine and catechin contents

In the measurement of catechin and caffeine content, ‘Seimei’, ‘Shunmei’, ‘Saemidori’, ‘Yabukita’, ‘Sayamamidori’, ‘Natsumidori’, ‘Fushun’ and ‘Asagiri’ were examined as Japanese green tea cultivars, and ‘Benifuki’, ‘Indo’, ‘Benikaori’ and ‘ShizuInzatsu131’ as other varieties. Shoots of cultivars grown in the same field with a bud and three leaves at the time of fourth or fifth leaf opening were handpicked at first crop in 2020. They were immediately steamed for 45 seconds to inactivate enzymes and dried to about 5% moisture content in a small (capacity, 50 g of leaves) tea rolling dryer (Terada Seisakusho, Shizuoka, Japan) and then a constant temperature blowing-type dryer (FV-1500; Advantec, Tokyo, Japan). All dried samples were powdered in a cyclone sample mill (UDY Co., Fort Collins, CO, USA), and were extracted as described previously (Maeda-Yamamoto et al. 2005). Caffeine and catechin contents were determined by a high-performance liquid chromatography as described previously (Monobe et al. 2019).

## Supplementary Material

pcae060_Supp

## Data Availability

The sequence data used in this article are available in the DNA Data Bank of Japan (DDBJ) Read Archive (DRA) under accession number DRA016707. The genome assembly and gene annotations have been deposited in DDBJ under the accession numbers BTIW01000001–BTIW01000181.
