# Supplementary material for: Chromosome-Scale Genome Assembly and Characterization of Top-Quality Japanese Green Tea Cultivar ‘Seimei’
Source: Plant Cell Physiol. 2024 May 27;65(8):1271–84. doi: 10.1093/pcp/pcae060 (PMC11369818; doi:10.1093/pcp/pcae060)
Supplement: pcae060_Supp [file pcae060_supp.zip › suppl_data/pcp-2024-e-00100-File007.pdf]

Supplementary Table S1. Sequence data for the genome assembly of 'Seimei'.

Data type	Tissue	DRA accession numbers
PacBio HiFi	Young shoots	DRR492905, DRR492906, DRR492907, DRR492908, DRR492909, DRR492910, DRR492911, DRR492912
Proximo HiC	Young shoots	DRR492915
IsoSeq	Young shoots	DRR492916, DRR492917
IsoSeq	Mature leaves	DRR492918
IsoSeq	Roots	DRR492919
RNA-Seq	Young shoots	DRR492920
RNA-Seq	Mature leaves	DRR492921
RNA-Seq	Roots	DRR492922

Supplementary Table S2. Statistics of chromosome and scaffold sequences.

Sequence ID	Length (bp)	# of AGTC	# of Ns	# of repeat-masked bases	% masked
Whole genome	3,157,427,346	3,157,407,346	20,000	2,505,524,114	79.4
Chr1	243,588,495	243,587,895	600	189,629,257	77.9
Chr2	225,099,778	225,098,978	800	177,143,417	78.7
Chr3	225,389,168	225,388,168	1,000	175,480,751	77.9
Chr4	248,952,868	248,951,168	1,700	194,809,306	78.3
Chr5	210,105,503	210,104,703	800	164,474,156	78.3
Chr6	221,661,953	221,659,253	2,700	171,739,705	77.5
Chr7	219,724,471	219,723,671	800	177,991,720	81.0
Chr8	214,857,925	214,856,325	1,600	171,993,680	80.1
Chr9	203,824,609	203,823,209	1,400	159,500,630	78.3
Chr10	193,918,808	193,917,408	1,400	154,684,758	79.8
Chr11	164,353,529	164,351,429	2,100	131,688,678	80.1
Chr12	174,263,572	174,262,572	1,000	138,734,898	79.6
Chr13	203,088,184	203,086,984	1,200	164,137,696	80.8
Chr14	164,769,179	164,767,979	1,200	127,685,224	77.5
Chr15	157,462,333	157,461,633	700	128,294,329	81.5
scaffold_1	20,753,668	20,753,668	0	20,385,531	98.2
scaffold_2	16,661,002	16,661,002	0	16,447,544	98.7
scaffold_3	16,479,734	16,479,734	0	16,072,877	97.5
scaffold_4	2,318,177	2,318,177	0	2,273,215	98.1
scaffold_5	2,155,776	2,155,776	0	2,155,624	100.0
scaffold_6	1,742,554	1,742,554	0	1,573,469	90.3
scaffold_7	1,624,188	1,623,688	500	1,507	0.1
scaffold_8	1,481,455	1,481,455	0	4,307	0.3
scaffold_9	1,475,775	1,475,775	0	1,449,036	98.2
scaffold_10	1,379,172	1,379,172	0	1,359,656	98.6
scaffold_11	1,128,339	1,128,339	0	1,103,201	97.8
scaffold_12	863,412	863,412	0	844,286	97.8
scaffold_13	764,628	764,628	0	754,026	98.6
scaffold_14	752,086	752,086	0	737,294	98.0
scaffold_15	698,238	698,238	0	688,983	98.7
scaffold_16	683,633	683,633	0	674,621	98.7
scaffold_17	596,786	596,786	0	152	0.0
scaffold_18	524,261	524,261	0	518,594	98.9
scaffold_19	490,704	490,704	0	480,875	98.0
scaffold_20	417,451	417,451	0	412,278	98.8

Sequence ID	Length (bp)	# of AGTC	# of Ns	# of repeat-masked bases	% masked
scaffold_21	416,250	416,250	0	408,161	98.1
scaffold_22	382,471	382,471	0	374,568	97.9
scaffold_23	364,818	364,818	0	364,812	100.0
scaffold_24	363,891	363,891	0	0	0.0
scaffold_25	354,772	354,772	0	345,787	97.5
scaffold_26	349,832	349,832	0	336,162	96.1
scaffold_27	330,285	330,285	0	323,305	97.9
scaffold_28	283,068	283,068	0	276,995	97.9
scaffold_29	282,265	282,265	0	277,473	98.3
scaffold_30	278,296	278,296	0	267,168	96.0
scaffold_31	251,042	251,042	0	3,214	1.3
scaffold_32	245,348	245,348	0	87	0.0
scaffold_33	243,220	243,220	0	238,619	98.1
scaffold_34	240,748	240,748	0	236,346	98.2
scaffold_35	237,424	237,424	0	233,570	98.4
scaffold_36	235,598	235,598	0	53,143	22.6
scaffold_37	220,615	220,615	0	217,663	98.7
scaffold_38	211,673	211,673	0	207,036	97.8
scaffold_39	209,585	209,585	0	202,812	96.8
scaffold_40	209,354	209,354	0	205,464	98.1
scaffold_41	208,702	208,702	0	204,450	98.0
scaffold_42	205,179	205,179	0	199,508	97.2
scaffold_43	204,391	204,391	0	197,922	96.8
scaffold_44	192,230	192,230	0	192,195	100.0
scaffold_45	192,158	192,158	0	188,332	98.0
scaffold_46	189,464	189,464	0	187,125	98.8
scaffold_47	154,064	154,064	0	152,375	98.9
scaffold_48	151,135	151,135	0	148,793	98.5
scaffold_49	150,812	150,812	0	148,476	98.5
scaffold_50	148,674	148,674	0	3,770	2.5
scaffold_51	143,758	143,758	0	142,772	99.3
scaffold_52	142,775	142,775	0	140,654	98.5
scaffold_53	139,830	139,830	0	135,155	96.7
scaffold_54	135,710	135,710	0	30,648	22.6
scaffold_55	131,829	131,829	0	129,568	98.3
scaffold_56	127,827	127,827	0	126,558	99.0
scaffold_57	125,405	125,405	0	28,736	22.9

Sequence ID	Length (bp)	# of AGTC	# of Ns	# of repeat-masked bases	% masked
scaffold_58	123,386	123,386	0	119,334	96.7
scaffold_59	119,455	119,455	0	0	0.0
scaffold_60	118,866	118,866	0	115,683	97.3
scaffold_61	118,099	118,099	0	117,138	99.2
scaffold_62	115,222	115,222	0	113,595	98.6
scaffold_63	114,548	114,548	0	110,612	96.6
scaffold_64	105,284	105,284	0	99,185	94.2
scaffold_65	99,351	99,351	0	22,442	22.6
scaffold_66	97,770	97,770	0	96,641	98.9
scaffold_67	97,436	97,436	0	97,269	99.8
scaffold_68	83,066	83,066	0	80,614	97.1
scaffold_69	76,646	76,646	0	75,284	98.2
scaffold_70	74,626	74,626	0	72,275	96.9
scaffold_71	71,174	71,174	0	3,913	5.5
scaffold_72	71,117	71,117	0	70,003	98.4
scaffold_73	65,915	65,415	500	280	0.4
scaffold_74	64,660	64,660	0	5,165	8.0
scaffold_75	63,718	63,718	0	62,982	98.8
scaffold_76	63,533	63,533	0	63,525	100.0
scaffold_77	62,195	62,195	0	3,012	4.8
scaffold_78	59,840	59,840	0	59,002	98.6
scaffold_79	59,259	59,259	0	3,924	6.6
scaffold_80	58,691	58,691	0	57,900	98.7
scaffold_81	58,428	58,428	0	58,422	100.0
scaffold_82	58,033	58,033	0	3,019	5.2
scaffold_83	56,496	56,496	0	55,869	98.9
scaffold_84	55,097	55,097	0	54,986	99.8
scaffold_85	51,591	51,591	0	51,249	99.3
scaffold_86	51,320	51,320	0	3,636	7.1
scaffold_87	51,029	51,029	0	50,514	99.0
scaffold_88	49,527	49,527	0	48,611	98.2
scaffold_89	48,920	48,920	0	2,172	4.4
scaffold_90	47,245	47,245	0	479	1.0
scaffold_91	46,773	46,773	0	46,772	100.0
scaffold_92	46,096	46,096	0	0	0.0
scaffold_93	45,922	45,922	0	549	1.2
scaffold_94	45,895	45,895	0	45,543	99.2

Sequence ID	Length (bp)	# of AGTC	# of Ns	# of repeat-masked bases	% masked
scaffold_95	44,769	44,769	0	44,141	98.6
scaffold_96	44,546	44,546	0	2,880	6.5
scaffold_97	44,494	44,494	0	3,169	7.1
scaffold_98	44,372	44,372	0	413	0.9
scaffold_99	44,333	44,333	0	3,851	8.7
scaffold_100	42,320	42,320	0	3,637	8.6
scaffold_101	42,125	42,125	0	413	1.0
scaffold_102	41,478	41,478	0	413	1.0
scaffold_103	41,157	41,157	0	40,482	98.4
scaffold_104	41,091	41,091	0	41,091	100.0
scaffold_105	39,659	39,659	0	39,658	100.0
scaffold_106	39,239	39,239	0	38,604	98.4
scaffold_107	38,751	38,751	0	323	0.8
scaffold_108	38,640	38,640	0	37,747	97.7
scaffold_109	38,086	38,086	0	3,445	9.1
scaffold_110	38,038	38,038	0	0	0.0
scaffold_111	37,595	37,595	0	323	0.9
scaffold_112	36,836	36,836	0	289	0.8
scaffold_113	36,368	36,368	0	32,587	89.6
scaffold_114	35,792	35,792	0	2,740	7.7
scaffold_115	34,527	34,527	0	34,432	99.7
scaffold_116	34,507	34,507	0	289	0.8
scaffold_117	34,371	34,371	0	515	1.5
scaffold_118	34,081	34,081	0	3,459	10.2
scaffold_119	33,850	33,850	0	3,442	10.2
scaffold_120	33,709	33,709	0	289	0.9
scaffold_121	33,464	33,464	0	3,321	9.9
scaffold_122	33,431	33,431	0	0	0.0
scaffold_123	32,191	32,191	0	289	0.9
scaffold_124	31,835	31,835	0	2,886	9.1
scaffold_125	31,653	31,653	0	289	0.9
scaffold_126	31,393	31,393	0	1,038	3.3
scaffold_127	31,316	31,316	0	289	0.9
scaffold_128	31,061	31,061	0	319	1.0
scaffold_129	30,974	30,974	0	515	1.7
scaffold_130	30,800	30,800	0	1,774	5.8
scaffold_131	30,757	30,757	0	289	0.9

Sequence ID	Length (bp)	# of AGTC	# of Ns	# of repeat-masked bases	% masked
scaffold_132	30,666	30,666	0	515	1.7
scaffold_133	30,297	30,297	0	30,249	99.8
scaffold_134	30,089	30,089	0	515	1.7
scaffold_135	29,656	29,656	0	29,656	100.0
scaffold_136	29,480	29,480	0	289	1.0
scaffold_137	29,324	29,324	0	0	0.0
scaffold_138	28,803	28,803	0	18,238	63.3
scaffold_139	28,728	28,728	0	26,678	92.9
scaffold_140	28,209	28,209	0	19,847	70.4
scaffold_141	28,075	28,075	0	25,979	92.5
scaffold_142	26,949	26,949	0	354	1.3
scaffold_143	26,832	26,832	0	24,736	92.2
scaffold_144	26,577	26,577	0	289	1.1
scaffold_145	26,487	26,487	0	0	0.0
scaffold_146	26,408	26,408	0	2,973	11.3
scaffold_147	26,308	26,308	0	289	1.1
scaffold_148	25,726	25,726	0	25,723	100.0
scaffold_149	25,174	25,174	0	3,321	13.2
scaffold_150	25,173	25,173	0	3,321	13.2
scaffold_151	25,156	25,156	0	2,975	11.8
scaffold_152	25,081	25,081	0	24,936	99.4
scaffold_153	24,994	24,994	0	3,321	13.3
scaffold_154	23,933	23,933	0	23,885	99.8
scaffold_155	22,636	22,636	0	22,588	99.8
scaffold_156	22,596	22,596	0	289	1.3
scaffold_157	22,424	22,424	0	22,282	99.4
scaffold_158	22,396	22,396	0	22,348	99.8
scaffold_159	20,123	20,123	0	20,117	100.0
scaffold_160	19,461	19,461	0	19,383	99.6
scaffold_161	19,179	19,179	0	19,125	99.7
scaffold_162	18,034	18,034	0	17,983	99.7
scaffold_163	17,588	17,588	0	17,582	100.0
scaffold_164	17,547	17,547	0	17,493	99.7
scaffold_165	15,867	15,867	0	446	2.8
scaffold_166	15,620	15,620	0	446	2.9

Supplementary Table S3. Genome assembly and gene annotation of 'Seimei' and other tea cultivars.

	Seimei	DASZ (wild tea)	Nanyongensis (*C. oleifera*)
**Reference**	This study	Zhang et al., 2020	Lin et al., 2022
**Assemble methods**	PacBio HiFi, HiC	PacBio, HiC, Illumina	PacBio, HiC, 10X, Bionano
**Genome size (Gb)**	3.2	3.1	2.9
**Size of 15 chromosomes (Gb)**	3.1	3.1	2.6
**Number of scaffolds**	181	1,231	2,143
**Scaffold N50 (Mb)**	214.9	204.2	185.4
**Non-ATGC bases (Mb)**	0.2	1.6	254.4
**Number of gaps**	293	4,209	5,223
**BUSCO (Genome)**			
**Complete (Single, Dups.)**	94.8% (88.4%, 6.4%)	94.8% (88.7%, 6.1%)	92.2% (84.5%, 7.7%)
**Fragmented**	2.0%	2.1%	2.1%
**Missing**	3.2%	3.1%	5.7%
**GC content (%)**	38.8	39.0	37.5
**LTR assembly index**	14.2	14.8	16.3
**Repetitive bases (Gb, %)**	2.51 (79.4%)	2.47 (79.32%)	1.95 (67.30%)
**Number of loci**	55,235	33,021	42,462
**Number of transcripts**	91,390	33,021	46,612
**BUSCO (Protein)**			
**Complete (Single, Dups.)**	97.5% (52.3%, 45.2%)	83.3% (77.8%, 5.5%)	52.0% (32.0%, 20.0%)
**Fragmented**	1.5%	7.2%	5.5%
**Missing**	1.0%	9.5%	42.5%

Supplementary Table S4. Positions of markers on the genetic and physical maps.

Marker	Genetic map		Physical map (Seimei)		E-value	Identity
	Linkage group	Position on the linkage map	Chromosome	Position on the chromosome		
MSE0158	LG01	103.292	Chr4	243,520,037	0	97.83
MSE0039	LG01	94.869	Chr4	224,802,055	1E-121	91.3
MSG0361	LG01	92.07	Chr4	219,413,434	0	90.56
MSG0258	LG01	81.547	Chr4	205,724,515	0	92.47
MSE0269	LG01	74.416	Chr4	195,493,430	0	96.86
A10	LG01	61.913	Chr4	172,938,773	0	97.49
MSE0282	LG01	54.366	Chr4	162,658,889	0	96.98
MSE0054	LG01	51.759	Chr4	117,940,325	1E-135	99.63
MSG0835	LG01	56.049	Chr4	155,758,466	0	97.5
MSG0542	LG01	51.198	Chr4	158,728,302	0	94.58
MSG0811	LG01	52.441	Chr4	148,606,479	0	92.94
MSG0814	LG01	46.551	Chr4	110,010,810	0	94.25
MSG0318	LG01	42.533	Chr4	95,482,761	0	95.68
MSG0274	LG01	39.876	Chr4	84,834,497	0	99.84
MSG0188	LG01	38.408	Chr4	81,133,510	3E-175	91.86
MSE0289	LG01	24.338	Chr4	49,435,374	0	98.78
MSG0740	LG01	4.775	Chr4	19,985,773	0	98.06
MSG0460	LG02	0	Chr6	6,578,509	0	99.64
MSE0177	LG02	3.114	Chr6	5,193,628	0	99.27
MSE0207	LG02	16.501	Chr6	35,634,702	0	98.91
MSG0471	LG02	20.818	Chr6	39,334,096	0	94.93
MSG0544	LG02	25.342	Chr6	50,409,406	0	98.13
MSE0173	LG02	30.274	Chr6	59,130,794	0	98.7
MSG0429	LG02	34.935	Chr6	60,968,246	0	98.73
MSE0309	LG02	38.387	Chr6	66,778,373	1E-117	91.72
CsFM1103	LG02	37.525	Chr8	213,247,773	0	99.91
MSG0655	LG02	64.336	Chr6	140,333,174	0	93.44
MSE0263	LG02	72.723	Chr6	161,117,152	0	99.85
MSG0609	LG02	78.7	Chr6	179,076,110	0	98.06
MSG0518	LG02	83.912	Chr6	193,145,446	0	96.99
MSE0023	LG02	87.772	Chr6	195,755,724	0	100
MSE0305	LG02	93.855	Chr6	209,507,236	0	97.49
MSE0351	LG02	94.421	Chr6	210,098,180	8E-78	97.7
CsFM1094	LG02	96.917	Chr6	213,008,822	0	96.79
MSE0223	LG03	1.945	Chr1	3,309,786	5E-150	95.45

Marker	Genetic map		Physical map (Seimei)		E-value	Identity
	Linkage group	Position on the linkage map	Chromosome	Position on the chromosome		
MSE0231	LG03	5.491	Chr15	19,509,089	0	97.84
CsFM1619	LG03	6.818	Chr1	19,697,809	0	97.59
MSE0045	LG03	6.241	Chr1	20,136,091	7E-146	96.83
MSE0245	LG03	11.586	Chr1	24,002,044	9E-150	99.34
MSG0371	LG03	15.87	Chr1	26,671,190	0	99.22
MSE0063	LG03	21.207	Chr1	37,617,630	0	98.12
MSE0006	LG03	21.207	Chr1	37,617,630	0	97.65
MSG0219	LG03	28.887	Chr1	74,230,944	0	99.28
MSG0572	LG03	34.754	Chr1	93,757,567	0	97
MSG0592	LG03	38.835	Chr1	103,168,398	0	97.36
MSG0007	LG02	95.238	Chr1	111,657,025	0	97.02
MSG0533	LG03	47.96	Chr1	126,100,998	0	98.33
MSG0382	LG03	46.426	Chr1	121,295,995	0	100
MSG0255	LG03	48.757	Chr1	121,875,367	0	100
MSG0322	LG03	56.923	Chr1	141,974,694	0	94.41
CsFM1599	LG03	60.931	Chr1	148,610,895	3E-82	92.23
MSE0082	LG03	66.251	Chr1	155,502,397	7E-151	95.77
MSE0029	LG03	66.932	Chr1	164,216,724	3E-105	100
MSG0717	LG03	75.303	Chr1	181,578,146	0	96.47
MSE0065	LG03	87.68	Chr1	199,392,118	3E-113	99.15
MSE0244	LG03	96.259	Chr1	214,409,191	0	99.67
MSE0196	LG03	99.929	Chr1	219,066,776	6E-154	100
MSE0061	LG03	98.29	Chr1	219,066,776	6E-154	100
MSG0612	LG03	104.033	Chr1	221,836,973	0	95.03
MSE0250	LG04	0	Chr5	8,227,813	0	94.99
MSE0226	LG04	0.867	Chr5	8,406,318	0	98.72
MSE0336	LG04	8.118	Chr5	10,283,126	0	97.73
MSG0742	LG04	6.834	Chr5	16,111,950	0	99.12
MSG0530	LG04	19.635	Chr5	35,650,107	0	100
MSG0598	LG04	20.376	Chr5	36,118,858	0	98.3
MSE0003	LG04	22.796	Chr5	44,680,901	0	90.57
MSG0672	LG04	16.934	Chr12	149,140,192	1E-105	96.6
MSG0162	LG04	26.278	Chr5	52,068,320	0	99.7
MSG0312	LG04	39.729	Chr5	95,844,982	0	93.98
MSG0673	LG04	37.842	Chr5	89,409,342	0	98.3
MSG0444	LG04	56.228	Chr5	104,715,589	0	92.76

Marker	Genetic map		Physical map (Seimei)		E-value	Identity
	Linkage group	Position on the linkage map	Chromosome	Position on the chromosome		
MSG0213	LG04	46.086	Chr5	119,071,906	0	97.02
MSG0400	LG04	47.997	Chr5	126,700,055	0	99.48
MSG0236	LG04	49.855	Chr5	124,039,460	0	99.63
MSG0642	LG04	93.194	Chr5	202,476,720	0	97.45
MSG0380	LG04	90.304	Chr5	198,654,528	0	99.73
MSE0204	LG05	0	Chr8	33,636,060	6E-157	98.76
MSE0296	LG05	23.61	Chr8	25,696,080	6E-132	98.54
MSE0299	LG05	23.61	Chr8	30,936,831	0	98.15
MSE0156	LG05	25.388	Chr8	40,841,324	0	99.02
MSE0053	LG05	26.989	Chr8	48,135,014	0	99.52
MSE0330	LG05	44.156	Chr8	60,433,629	0	100
MSG0541	LG05	80.499	Chr8	125,876,065	0	96.33
MSG0788	LG05	82.211	Chr8	135,097,503	0	95.9
MSG0571	LG05	85.692	Chr8	152,253,972	0	96.38
MSG0017	LG05	84.939	Chr8	153,098,065	0	94.53
MSG0702	LG05	88.085	Chr8	157,475,845	0	95.19
MSG0578	LG05	95.641	Chr8	168,494,434	0	96.39
MSE0167	LG06	90.648	Chr9	160,560,589	5E-91	97.98
MSG0096	LG06	91.346	Chr3	206,264,286	0	95.8
MSE0001	LG06	80.5	Chr3	195,104,376	0	92.65
MSG0308	LG06	71.927	Chr3	157,326,065	0	98.15
MSG0290	LG06	69.883	Chr3	151,277,683	0	90.68
MSG0482	LG06	68.046	Chr3	153,639,069	0	95.06
MSG0420	LG06	65.773	Chr3	143,850,677	0	95.53
MSG0563	LG06	57.195	Chr3	98,918,717	4E-175	93.67
MSG0713	LG06	57.875	Chr3	107,765,774	0	97.19
MSE0121	LG06	51.401	Chr3	80,902,076	4E-149	97.77
MSG0818	LG06	53.709	Chr3	80,382,407	0	95.17
MSG0706	LG06	49.289	Chr3	74,405,613	0	93.98
MSG0398	LG06	48.613	Chr3	76,610,643	0	98.36
CsFM1719	LG06	43.591	Chr5	204,650,813	3E-166	99.1
MSG0395	LG06	44.888	Chr3	70,316,791	0	97.36
MSG0532	LG06	42.536	Chr3	68,851,987	0	97.82
MSE0047	LG06	37.36	Chr3	51,818,388	0	100
MSE0035	LG06	27.508	Chr3	43,372,619	0	98.79
MSG0679	LG06	29.029	Chr3	39,371,222	0	98.53

Marker	Genetic map		Physical map (Seimei)		E-value	Identity
	Linkage group	Position on the linkage map	Chromosome	Position on the chromosome		
MSG0800	LG06	29.656	Chr3	40,314,598	0	96.33
MSG0233	LG06	29.656	Chr3	39,621,199	0	95.27
MSG0472	LG06	26.279	Chr3	32,505,264	0	96.77
MSE0072	LG06	26.026	Chr3	32,324,593	9E-87	100
MSG0311	LG06	19.149	Chr3	30,066,222	0	95.8
TUGMS92	LG06	16.421	Chr3	23,606,598	0	95.31
MSG0560	LG06	10.443	Chr3	18,599,941	8E-157	94.83
MSG0201	LG06	4.58	Chr3	17,831,584	0	99.66
MSG0558	LG07	100.934	Chr13	196,728,252	0	100
MSE0162	LG02	45.723	Chr5	31,969,375	4E-133	97.54
MSE0190	LG07	89.526	Chr13	185,741,766	0	99.81
MSE0008	LG07	86.889	Chr13	185,742,212	1E-115	93.73
MSE0108	LG07	86.077	Chr13	175,921,499	0	100
MSG0744	LG07	79.102	Chr13	162,092,149	0	99.24
MSE0348	LG07	61.819	Chr13	125,917,321	1E-93	96.7
MSG0668	LG07	63.741	Chr13	129,781,935	0	97.55
MSG0403	LG07	59.891	Chr13	123,026,894	0	100
MSG0540	LG07	33.847	Chr13	81,985,157	0	93.72
CsFM1089	LG07	19.165	Chr13	1,245,494	0	93.53
MSG0330	LG08	9.964	Chr2	11,796,495	0	98.72
MSE0237	LG08	6.9	Chr2	3,313,098	0	100
CsL59	LG08	3.664	Chr2	891,788	0	93.61
CsL58	LG08	3.664	Chr2	1,424,904	0	95.58
MSG0033	LG08	23.704	Chr2	44,741,993	0	98.52
MSG0237	LG08	27.392	Chr2	51,250,530	0	93.02
MSG0221	LG08	28.302	Chr2	44,741,810	0	97.5
MSG0720	LG08	53.623	Chr2	92,031,932	0	97.79
CsL74	LG08	50.786	Chr2	94,887,498	0	97.32
MSE0100	LG08	58.114	Chr2	97,999,855	2E-140	97.33
MSE0135	LG08	56.332	Chr13	171,781,755	6E-108	98.68
MSG0661	LG08	59.439	Chr2	105,633,521	1E-131	97.17
MSE0271	LG08	60.363	Chr2	111,923,347	0	94.53
MSG0413	LG08	60.779	Chr2	120,146,378	0	93.04
MSG0821	LG08	70.81	Chr2	147,903,303	0	97.52
MSE0215	LG08	72.609	Chr2	157,438,296	1E-133	97.22
MSG0700	LG08	70.178	Chr2	151,834,016	0	99.84

Marker	Genetic map		Physical map (Seimei)		E-value	Identity
	Linkage group	Position on the linkage map	Chromosome	Position on the chromosome		
MSG0779	LG08	76.083	Chr2	171,965,416	0	97.99
MSE0320	LG08	77.724	Chr2	168,504,785	7E-127	99.23
MSG0142	LG08	77.109	Chr2	168,995,955	0	91.48
MSE0159	LG08	79.221	Chr2	174,757,916	6E-128	95.83
MSE0067	LG08	79.92	Chr2	192,563,037	6E-171	98.58
MSE0291	LG08	84.077	Chr2	197,989,356	0	97.38
CsL79	LG08	95.872	Chr2	205,598,552	0	95.37
MSG0388	LG08	89.505	Chr2	205,030,059	0	96.41
MSE0089	LG08	92.216	Chr2	208,428,390	4E-134	94.77
MSG0587	LG08	93.109	Chr2	209,061,927	0	98.87
TM064	LG08	106.212	Chr2	217,999,500	3E-134	98.22
MSG0731	LG09	0	Chr7	18,866,707	0	98.19
MSE0181	LG09	10.489	Chr7	28,496,516	0	99.31
MSE0260	LG09	17.121	Chr7	39,675,187	0	100
MSG0300	LG09	19.192	Chr7	45,429,295	0	91.35
MSG0426	LG09	16.066	Chr7	39,401,511	0	93.09
MSE0339	LG09	31.243	Chr7	68,272,970	1E-179	98.63
MSE0113	LG09	33.467	Chr7	72,409,348	2E-77	97.69
MSG0297	LG09	38.141	Chr7	74,161,655	0	98.05
MSG0760	LG09	42.665	Chr7	82,864,758	0	98.4
MSG0511	LG09	46.164	Chr7	97,231,582	0	93.84
MSG0574	LG09	46.164	Chr7	98,577,091	0	95.9
MSG0452	LG09	52.296	Chr7	128,757,905	0	93.45
MSG0657	LG09	54.298	Chr7	135,815,522	0	97.4
MSE0101	LG09	53.937	Chr7	130,275,171	0	97.36
MSE0306	LG09	58.005	Chr7	133,542,435	9E-116	100
MSG0577	LG09	62.497	Chr7	172,662,537	0	95.59
MSE0012	LG09	74.278	Chr7	188,479,363	1E-138	95.47
MSE0143	LG09	82.86	Chr7	206,562,733	1E-111	100
MSG0421	LG09	86.135	Chr7	209,475,388	0	92.2
MSG0652	LG10	86.442	Chr9	175,958,489	0	97.11
MSG0623	LG10	74.721	Chr9	144,204,426	0	93.66
MSG0688	LG10	73.748	Chr9	134,755,962	0	95.96
MSG0604	LG10	71.867	Chr9	131,962,800	0	98.01
MSG0594	LG10	63.232	Chr9	113,316,954	0	99.06
MSE0149	LG10	61.3	Chr9	113,010,589	3E-111	100

Marker	Genetic map		Physical map (Seimei)		E-value	Identity
	Linkage group	Position on the linkage map	Chromosome	Position on the chromosome		
MSG0538	LG10	55.498	Chr9	103,245,402	2E-157	99.37
MSE0202	LG10	48.251	Chr9	92,308,206	0	99.1
MSG0451	LG10	40.524	Chr9	65,041,495	8E-152	92.94
MSE0273	LG10	34.676	Chr9	47,579,830	0	99.34
MSE0327	LG10	12.447	Chr9	115,565,499	0	99.78
MSE0212	LG10	7.534	Chr9	7,779,789	0	99.74
CsL57	LG02	62.945	Chr9	1,417,662	0	97.93
MSG0505	LG11	98.307	Chr11	148,131,939	0	96.85
MSG0714	LG11	90.841	Chr11	147,936,191	0	96.38
MSG0617	LG11	76.711	Chr11	129,358,764	0	99.17
MSG0457	LG11	71.027	Chr11	122,457,742	0	100
MSG0083	LG11	63.438	Chr11	111,529,734	0	91.81
MSE0043	LG11	55.348	Chr11	102,351,858	0	99.29
MSE0334	LG11	48.506	Chr11	91,166,500	0	96.76
MSG0665	LG11	45.21	Chr11	89,142,662	0	99.35
MSE0107	LG11	40.868	Chr13	84,673,129	1E-86	98.93
MSG0734	LG11	31.027	Chr11	72,927,759	0	93.78
MSG0477	LG11	22.962	Chr11	62,146,580	0	93.39
MSG0753	LG11	1.747	Chr11	18,516,736	0	97.09
CsFM1595	LG11	0	Chr11	22,232,783	4E-101	98.16
MSE0335	LG12	64.86	Chr10	158,127,275	0	100
MSG0008	LG12	66.414	Chr10	158,223,549	0	94.63
MSE0332	LG12	62.942	Chr10	150,189,590	6E-145	93.18
MSE0161	LG12	52.371	Chr10	140,698,841	0	97.98
MSG0325	LG12	53.225	Chr10	139,239,349	0	98.8
MSG0674	LG12	51.948	Chr10	139,892,855	0	99.74
MSE0030	LG12	50.922	Chr10	139,238,902	3E-144	95.34
MSG0832	LG12	50.056	Chr10	136,909,883	0	99.31
MSG0534	LG12	48.596	Chr10	135,613,995	0	97.85
MSE0019	LG12	45.589	Chr10	129,409,662	0	98.83
MSG0126	LG12	45.551	Chr10	129,014,935	0	96.28
MSG0260	LG12	41.937	Chr10	118,260,148	0	94.73
MSE0083	LG12	41.442	Chr10	121,997,847	4E-157	99.68
MSG0796	LG12	39.321	Chr10	115,250,530	0	99.15
MSE0228	LG12	38.392	Chr10	115,486,128	5E-159	99.07
MSE0265	LG12	26.652	Chr10	63,804,005	2E-169	96.69

Marker	Genetic map		Physical map (Seimei)		E-value	Identity
	Linkage group	Position on the linkage map	Chromosome	Position on the chromosome		
MSG0023	LG12	21.75	Chr10	65,415,852	0	98.9
MSG0627	LG12	8.591	Chr10	30,441,225	0	98.48
MSG0794	LG12	8.139	Chr10	29,571,676	0	98.17
MSG0685	LG13	1.861	Chr14	394,855	0	93.94
MSG0708	LG13	0	Chr14	5,843,937	0	93.02
MSG0102	LG13	2.359	Chr14	4,795,855	0	97.67
MSE0169	LG13	2.359	Chr14	6,217,232	0	99.63
MSG0272	LG13	7.653	Chr14	20,608,125	0	96.67
CsFM1214	LG13	15.252	Chr14	43,587,284	0	100
MSG0795	LG13	15.632	Chr14	41,046,175	0	95.55
MSG0799	LG13	14.093	Chr14	35,521,104	0	95.53
MSE0070	LG13	19.235	Chr14	50,394,485	9E-125	98.84
MSG0470	LG13	39.934	Chr14	100,390,608	0	96.04
MSG0271	LG13	42.816	Chr14	97,786,690	0	94.68
MSG0765	LG13	53.775	Chr14	122,109,042	0	92.4
MSG0385	LG13	60.249	Chr14	135,981,739	0	98.24
MSE0140	LG14	5.654	Chr12	11,725,561	0	99.81
MSG0637	LG14	7.373	Chr12	21,655,011	0	98.51
MSG0393	LG14	7.373	Chr12	20,888,780	0	91.62
MSG0660	LG14	9.544	Chr12	42,228,572	0	91.57
MSE0259	LG14	10.681	Chr12	43,844,424	0	100
MSG0234	LG14	12.841	Chr12	49,025,794	0	92.91
MSG0763	LG14	17.299	Chr12	60,662,741	0	93.57
MSG0523	LG14	25.504	Chr12	90,033,388	6E-171	96.97
MSG0693	LG14	29.238	Chr12	94,319,715	0	91.9
MSG0436	LG14	30.428	Chr12	93,912,562	0	94.25
MSG0401	LG14	37.577	Chr12	98,661,992	0	91.25
MSG0263	LG14	47.373	Chr12	117,864,658	0	97.44
MSG0607	LG14	51.049	Chr12	130,466,222	0	95.56
MSE0062	LG14	53.127	Chr12	134,931,761	0	99.51
MSG0570	LG14	55.019	Chr12	142,547,407	0	96.58
MSG0407	LG14	71.003	Chr12	159,748,652	0	94.83
MSG0812	LG14	75.619	Chr12	171,069,741	0	99.37
MSE0213	LG14	77.508	Chr12	171,059,860	0	99.76
CsFM1207	LG15	50.809	Chr15	115,069,818	9E-103	96.51
MSG0581	LG04	7.724	Chr15	108,254,523	0	92.02

Marker	Genetic map		Physical map (Seimei)		E-value	Identity
	Linkage group	Position on the linkage map	Chromosome	Position on the chromosome		
MSG0755	LG15	28.314	Chr15	63,503,905	0	95.71
MSG0699	LG15	4.283	Chr15	10,933,026	0	97.99
MSG0344	LG15	0	Chr15	4,286,867	0	94.93

Supplementary Table S5. Statistics of repeat annotation for the tea cultivars.

	Seimei		DASZ (wild tea)		Nanyongensis (*C. oleifera*)	
	**Length (Mb)**	**% of genome**	**Length (Mb)**	**% of genome**	**Length (Mb)**	**% of genome**
Bases masked as repeats	2,505.5	79.4	2,469.4	79.3	1,945.6	67.3
Retroelements	1,106.8	35.1	11,440.7	36.8	866.9	30.0
SINEs	2.1	0.1	1.6	0.1	1.4	0.1
LINEs	39.9	1.3	40.9	1.3	38.6	1.3
LTR elements	1,064.9	33.7	1,101.5	35.4	826.9	28.6
Ty1/Copia	155.8	4.9	159.3	5.1	145.4	5.0
Gypsy/DIRS1	887.6	28.1	922.2	29.6	660.9	22.9
DNA transposons	57.9	1.8	62.0	2.0	59.3	2.1
Rolling circles	9.0	0.3	9.2	0.3	8.0	0.3
Unclassified	1,310.4	41.5	1,239.5	39.8	1,002.2	34.7
Total interspersed repeats	2,475.0	78.4	2,445.6	78.6	1,928.4	66.7
Small RNA	10.1	0.3	1.5	0.1	0.9	0.0
Satellites	12.1	0.4	13.7	0.4	8.8	0.3
Simple repeats	0.3	0.0	0.2	0.0	0.2	0.0

Supplementary Table S6. Functional assignment of annotated genes in the 'Seimei' genome.

	# of loci	%
All loci	55,235	–
have homologs in the 'Shuchazao' annotation	50,950	92.2
have homologs among *Camellia* proteins in UniProt	51,324	92.9
have homologs in SwissProt	31,142	56.4
have homologs in TrEMBL	52,135	94.4
have InterPro domains	38,955	70.5
have GO terms assigned by InterProScan	27,970	50.6
have KO terms assigned by BlastKOALA	12,577	22.8

Supplementary Table S7. Sequence data for genomic diversity analysis of tea cultivars and lines used in this study.

Name	Type[a]	Breeding organization	Data type	DRA accession #
Seimei	Cultivar (JP Green tea)	NARO	Illumina	DRR492923
Sayamakaori	Cultivar (Admixture)	Saitama Pref.	Illumina	DRR492924
KanaCk17	Germplasm (Others)	Not applicable	Illumina	DRR492925
Yabukita	Cultivar (JP Green tea)	Private breeder	Illumina	DRR492926
Fushun	Cultivar (JP Green tea)	NARO	Illumina	DRR492927
Saemidori	Cultivar (JP Green tea)	NARO	Illumina	DRR492928
S6	Breeding line (Admixture)	NARO	Illumina	DRR492929
ShizuInzatsu131	Breeding line (Others)	Shizuoka Pref.	Illumina	DRR492930
Z1	Breeding line (Admixture)	NARO	Illumina	DRR492931
ShizuZai16	Germplasm (Admixture)	Not applicable	Illumina	DRR492932
Benifuki	Cultivar (Others)	NARO	Illumina	DRR492933
Sayamamidori	Cultivar (JP Green tea)	Saitama Pref.	Illumina	DRR492934
Kuritawase	Cultivar (JP Green tea)	Private breeder	Illumina	DRR492935
Marishi	Cultivar (Admixture)	Private breeder	Illumina	DRR492936
Indo	Cultivar (Others)	Kagoshima Pref.	Illumina	DRR492937
Natsumidori	Cultivar (JP Green tea)	NARO	Illumina	DRR492938
Asagiri	Cultivar (JP Green tea)	Kyoto Pref.	Illumina	DRR492939
Benikaori	Cultivar (Others)	Kagoshima Pref.	Illumina	DRR492940
Shunmei	Cultivar (JP Green tea)	NARO	Illumina	DRR492941
Shisen10	Germplasm (Others)	Not applicable	DNBSEQ	DRR492942
MkCk32	Germplasm (Others)	Not applicable	DNBSEQ	DRR492943
MC30	Germplasm (Others)	Not applicable	DNBSEQ	DRR492944
MkCp2	Germplasm (Others)	Not applicable	DNBSEQ	DRR492945

Name	Type[a]	Breeding organization	Data type	DRA accession #
Shuchazao	Cultivar (Others)	Not applicable	Illumina	SRR11672557[b]
Longjing 43 (LJ43)	Cultivar (Others)	Not applicable	Illumina	ERR4371156-ERR4371182[c]
DASZ	Germplasm (Wild)	Not applicable	Illumina	SRR10695775-SRR10695776[d]

[a] The population name defined in this study are shown in parentheses.

[b] Xia et al., 2020

[c] Wang et al., 2020

[d] Zhang et al., 2020
