# Supplementary material for: Chromosome-Scale Genome Assembly and Characterization of Top-Quality Japanese Green Tea Cultivar ‘Seimei’
Source: Plant Cell Physiol. 2024 May 27;65(8):1271–84. doi: 10.1093/pcp/pcae060 (PMC11369818; doi:10.1093/pcp/pcae060)
Supplement: pcae060_Supp [file pcae060_supp.zip › suppl_data/pcp-2024-e-00100-File009.pdf]

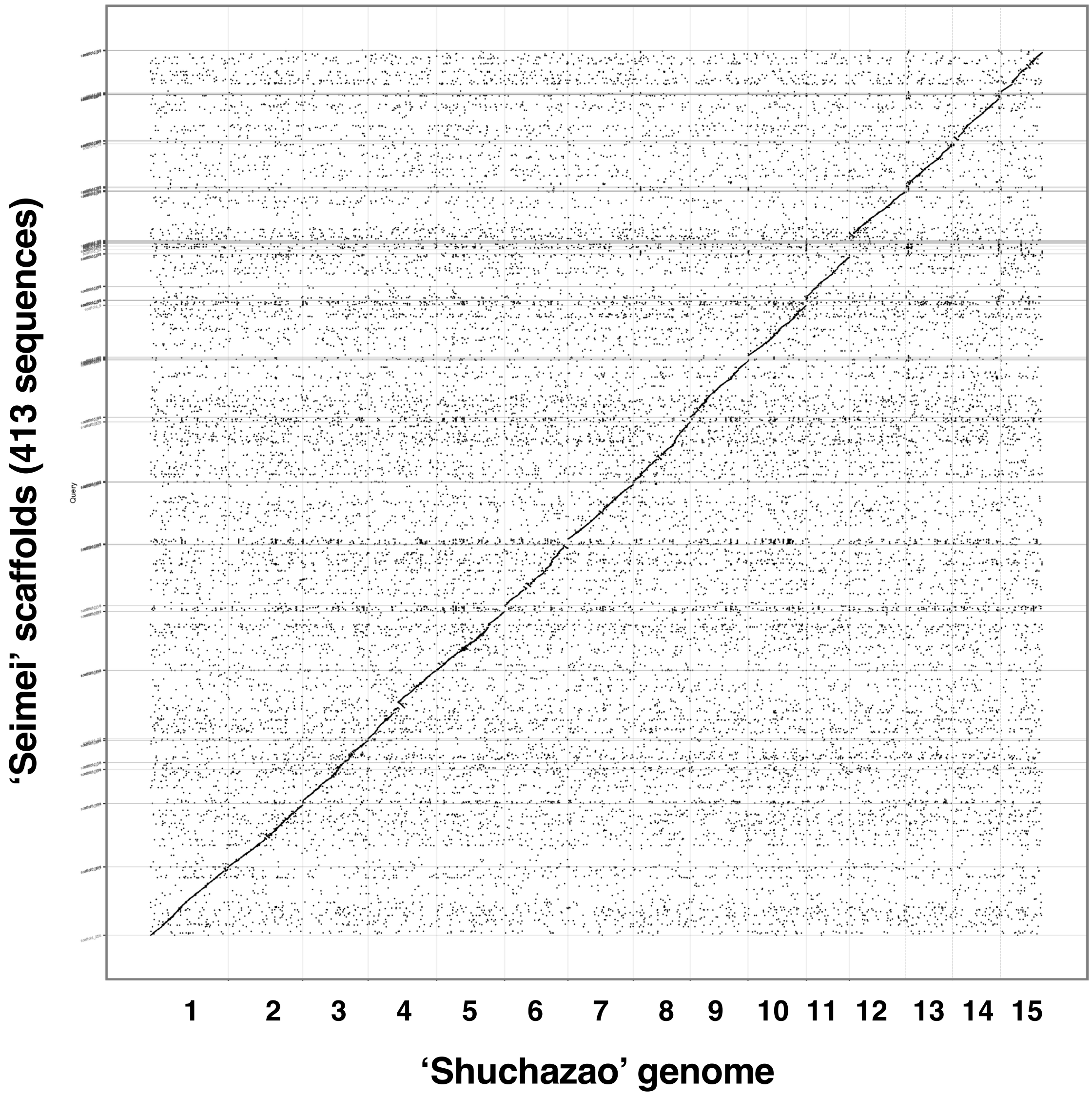

Supplementary Fig. S1 Dotplot between 413 scaffolds of 'Seimei' genome assembly and the 'Shuchazao' reference genome.

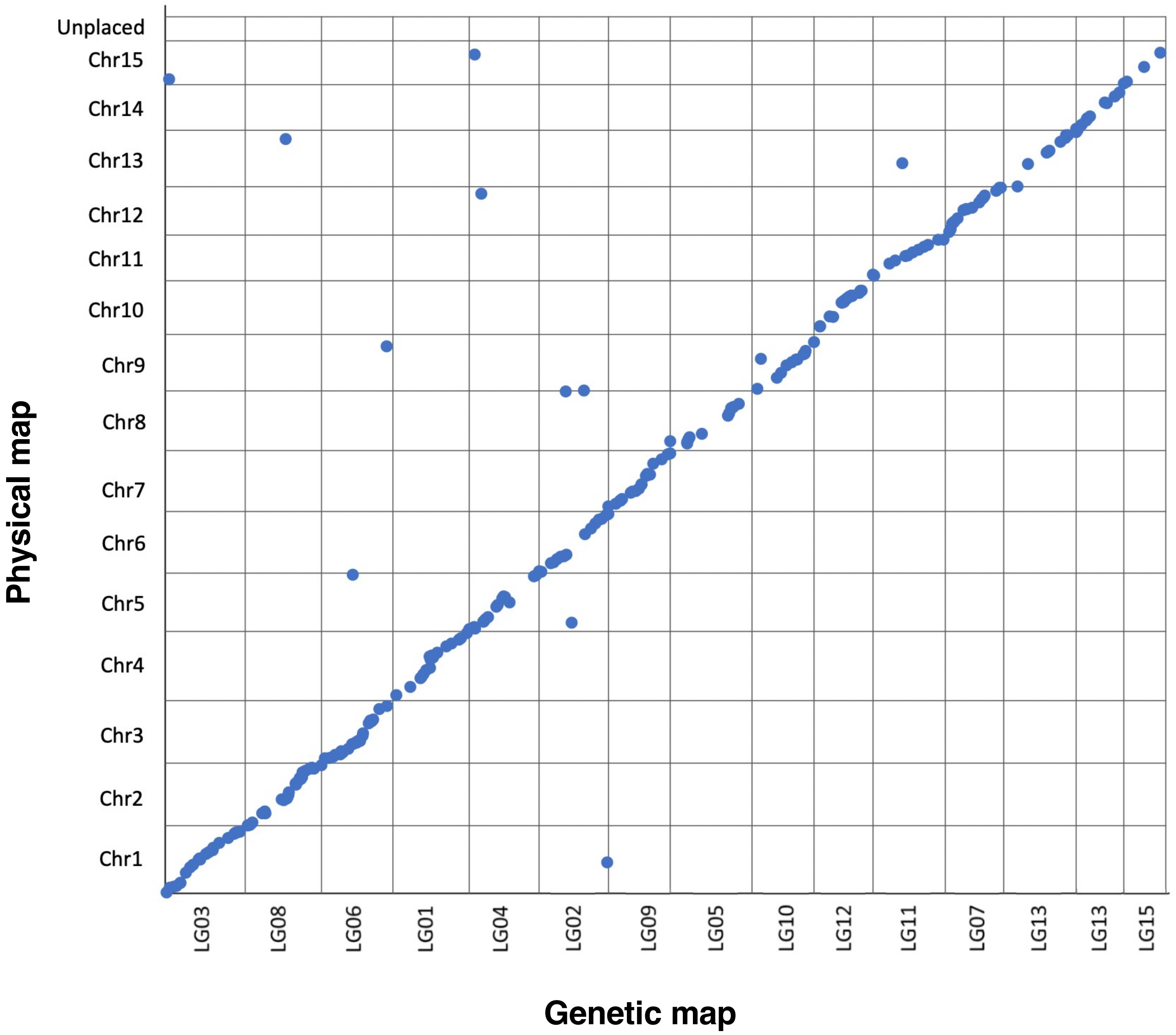

Supplementary Fig. S2 Correlation of genetic and physical maps. Physical positions of previously published markers in the 'Seimei' genome were plotted against their genetic positions on the linkage map.

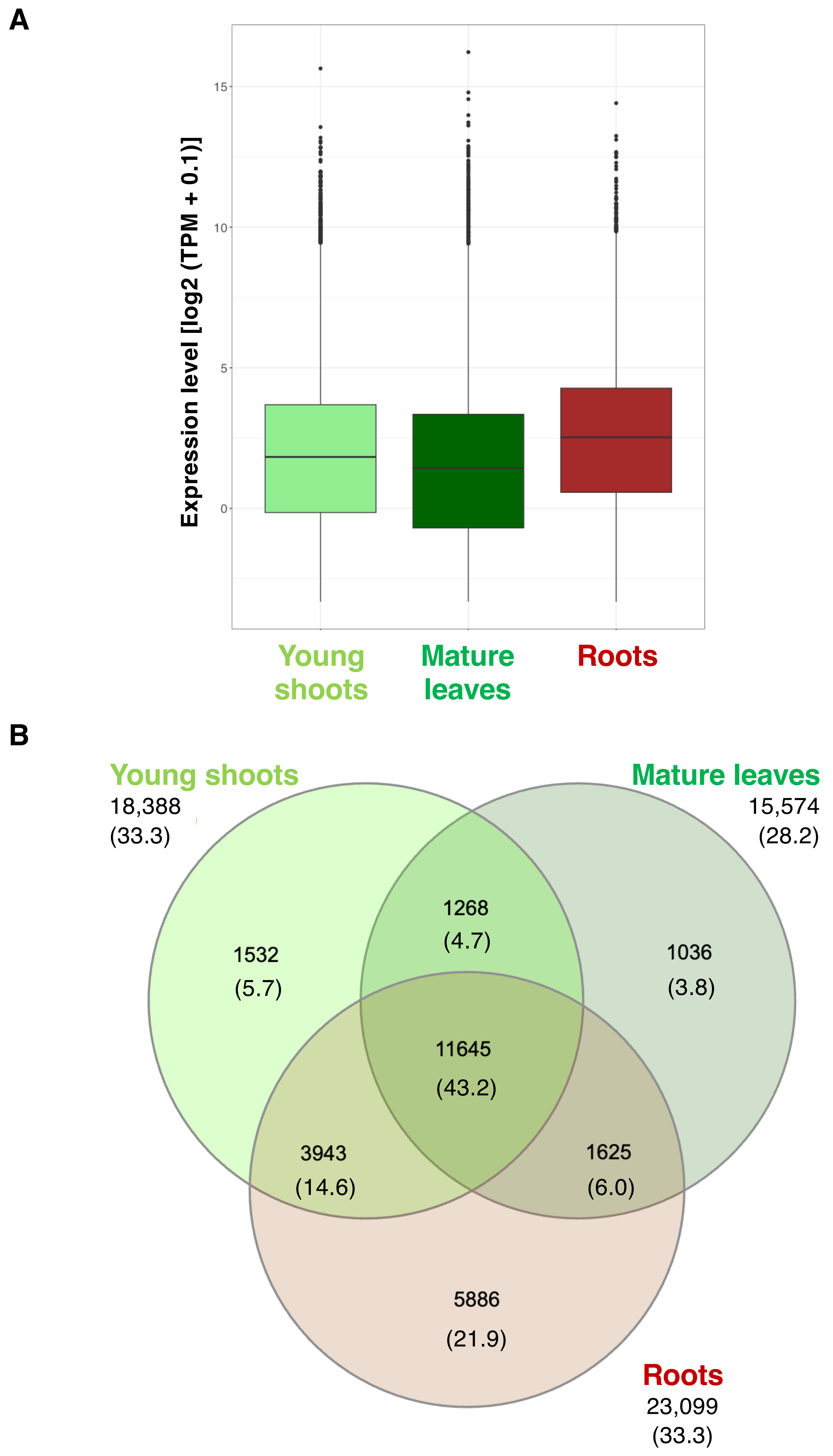

Supplementary Fig. S3 (A) Distributions of gene expression levels in three tissues. (B) A Venn diagram showing the numbers of genes expressed (TPM > 1) in each tissue. The values in parentheses are percentages of the total number of genes.

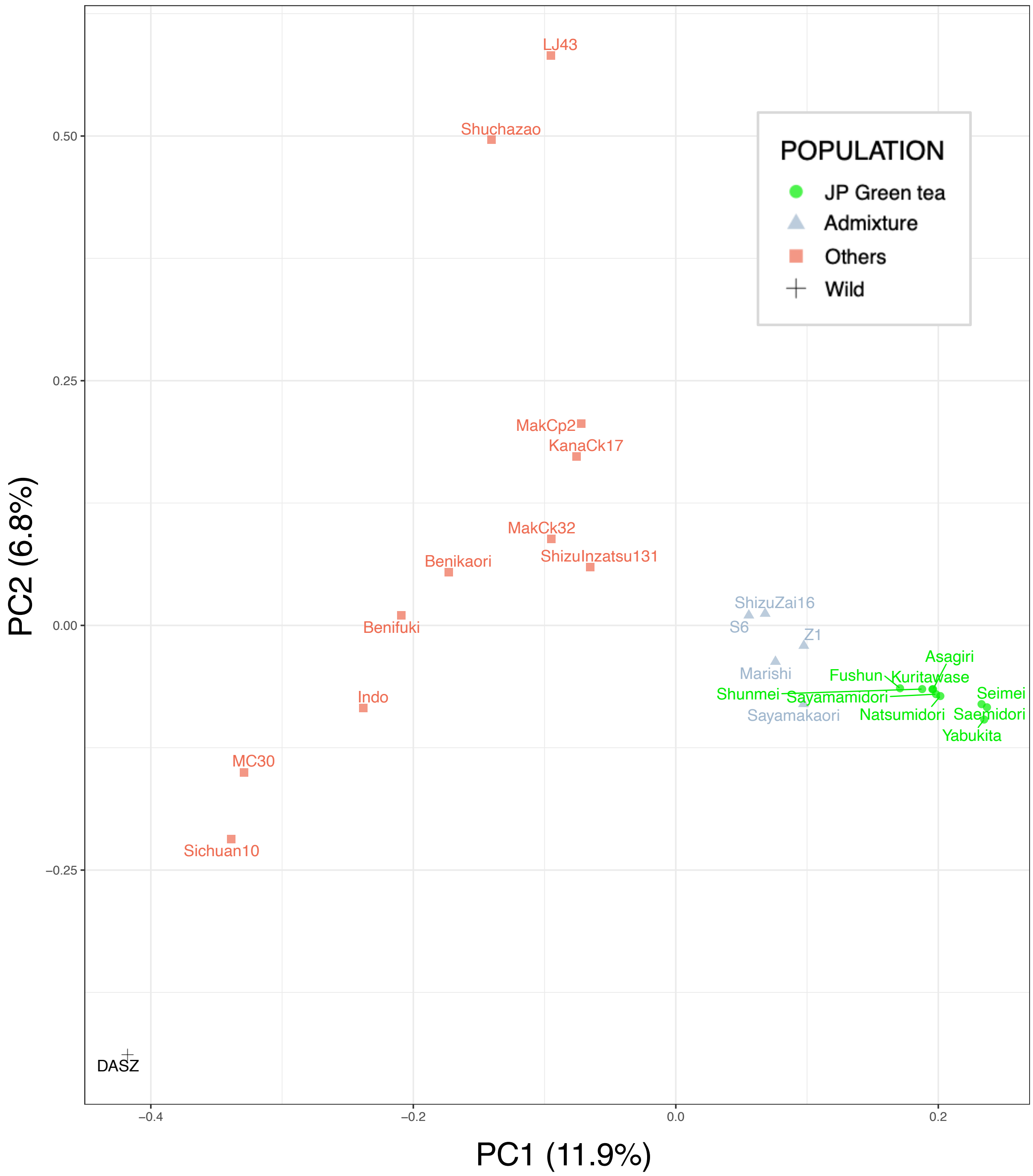

Supplementary Fig. S4 Principal component analysis of 26 tea varieties based on 33,616,569 bi-allelic and unlinked SNPs. Colors indicate the subpopulations defined in this study.

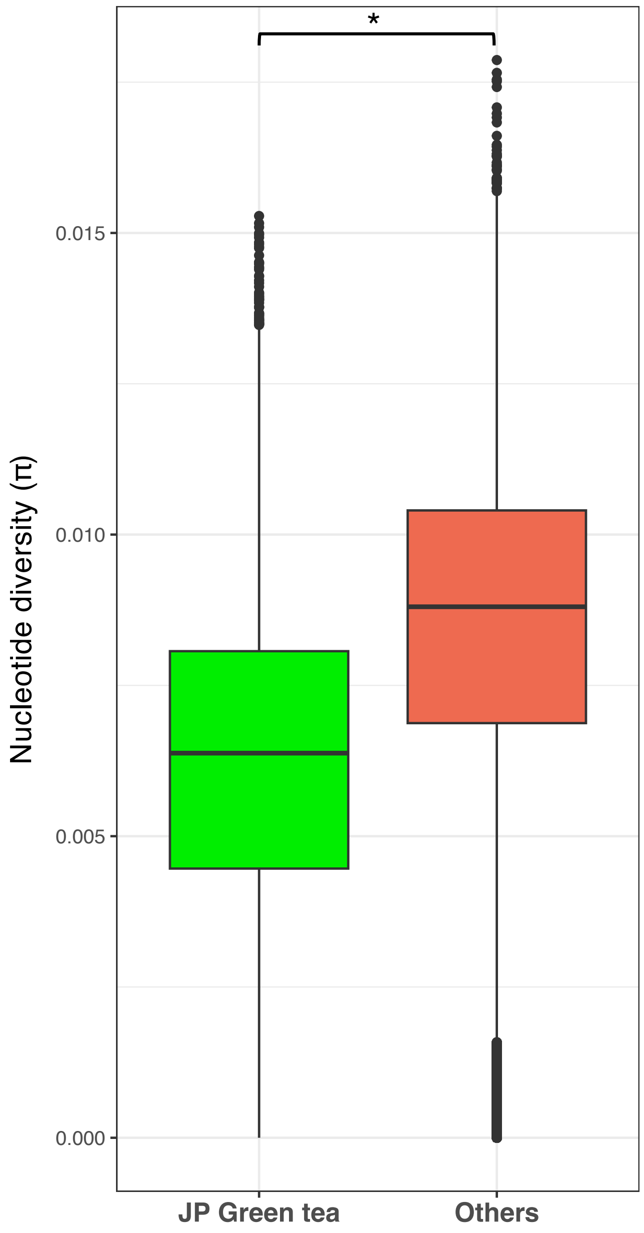

Supplementary Fig. S5 Nucleotide diversities (π) in Japanese green tea cultivars and other varieties. Significant differences: *P < 0.001 (Student's t-test using two-tailed distributions).

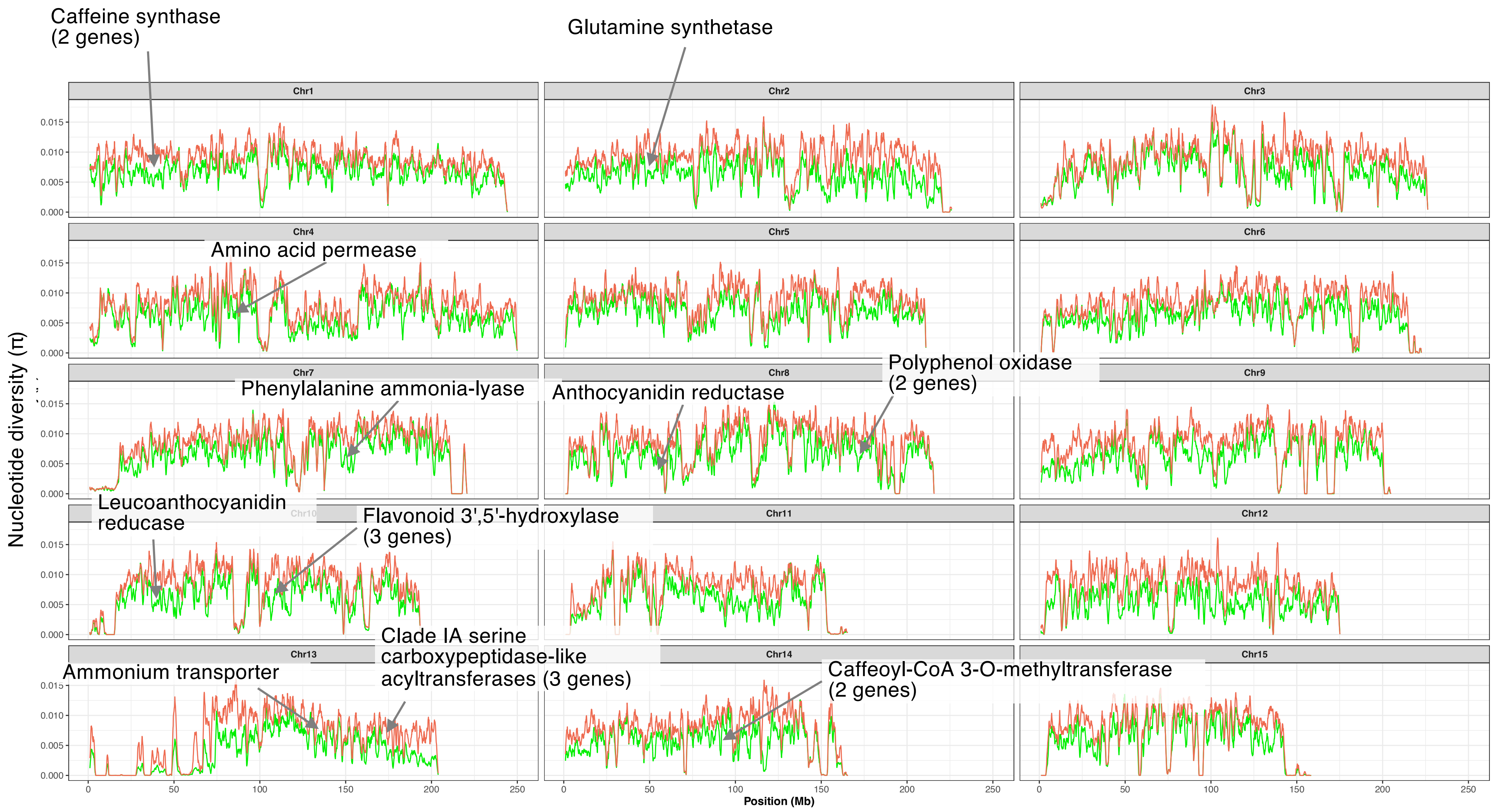

Supplementary Fig. S6 Distribution of nucleotide diversities in Japanese green tea cultivars (green) and other varieties (red). The positions of agronomically important genes are indicated.

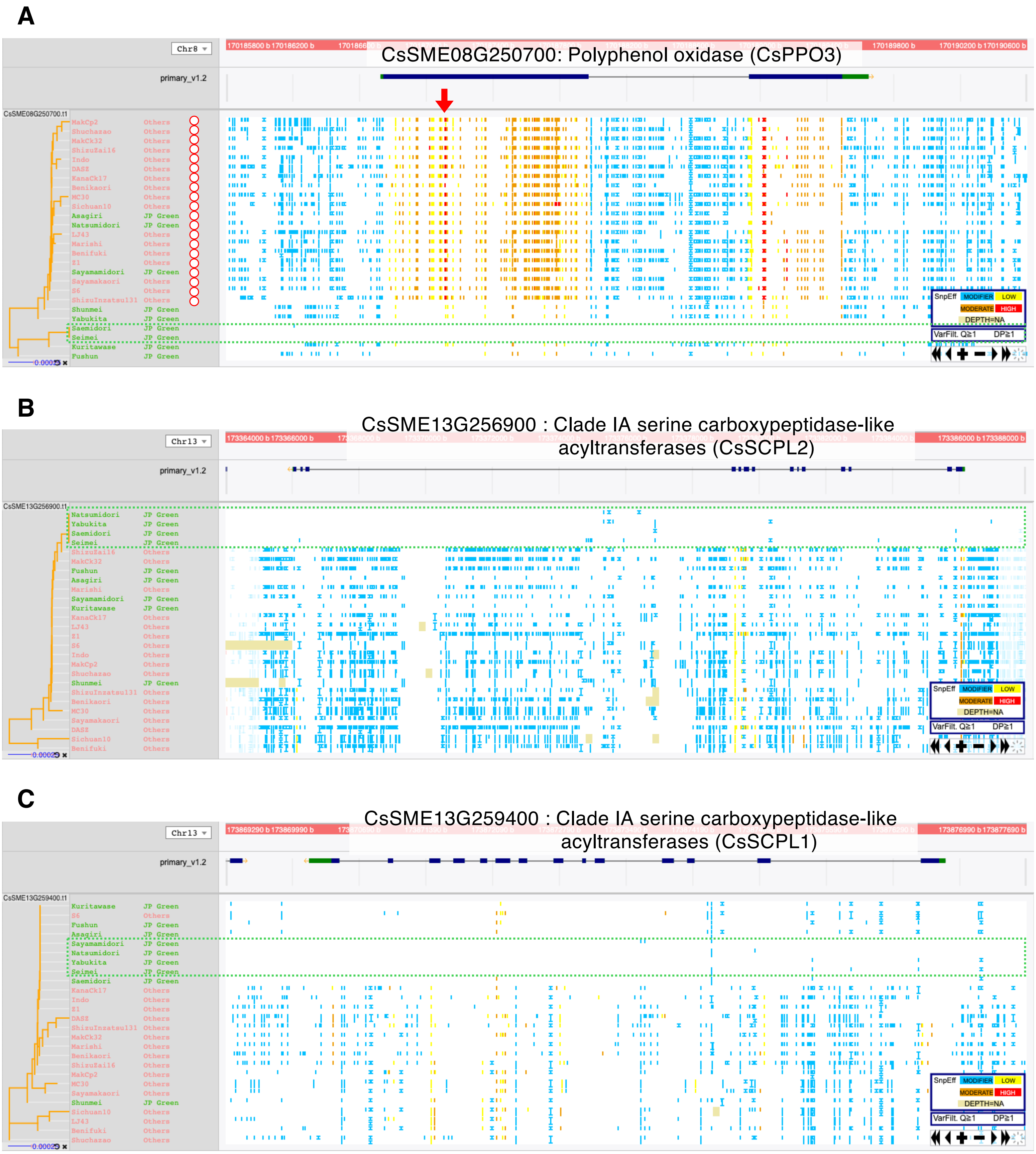

Supplementary Fig. S7 Nucleotide variations among 26 tea varieties around genes that might be related to characteristics of Japanese green tea cultivars. SNPs and InDels vs. the 'Seimei' genome are visualized using TASUKE+. Each variant is colored according to its effect on the protein sequence (from low to high). (A) Polyphenol oxidase (CsPPO3), (B, C) Clade IA serine carboxypeptidase-like acyltransferases (B) CsSCPL2 and (C) CsSCPL1. Green dotted rectangles indicate genomics regions fixed in homozygous 'Seimei'-type genotypes. Red arrow indicates a candidate functional mutation; red open circles indicate varieties heterozygous for the mutation.
